# Research Progress on Low-Pressure Powder Injection Molding

**DOI:** 10.3390/ma16010379

**Published:** 2022-12-30

**Authors:** Vahid Momeni, Margarete Hufnagl, Zahra Shahroodi, Joamin Gonzalez-Gutierrez, Stephan Schuschnigg, Christian Kukla, Clemens Holzer

**Affiliations:** 1 Polymer Processing, Montanuniversitaet Leoben, 8700 Leoben, Austria; 2 Functional Polymers Research Unit, Materials Research and Technology (MRT) Department, Luxembourg Institute of Science and Technology (LIST), L-4940 Luxembourg, Luxembourg; 3 Industrial Liaison Department, Montanuniversitaet Leoben, 8700 Leoben, Austria

**Keywords:** feedstock, debinding, sintering, binder system, thermal wick debinding, LPIM

## Abstract

Powder injection molding (PIM) is a well-known technique to manufacture net-shaped, complicated, macro or micro parts employing a wide range of materials and alloys. Depending on the pressure applied to inject the feedstock, this process can be separated into low-pressure (LPIM) and high-pressure (HPIM) injection molding. Although the LPIM and HPIM processes are theoretically similar, all steps have substantial differences, particularly feedstock preparation, injection, and debinding. After decades of focusing on HPIM, low-viscosity feedstocks with improved flowability have recently been produced utilizing low-molecular-weight polymers for LPIM. It has been proven that LPIM can be used for making parts in low quantities or mass production. Compared to HPIM, which could only be used for the mass production of metallic and ceramic components, LPIM can give an outstanding opportunity to cover applications in low or large batch production rates. Due to the use of low-cost equipment, LPIM also provides several economic benefits. However, establishing an optimal binder system for all powders that should be injected at extremely low pressures (below 1 MPa) is challenging. Therefore, various defects may occur throughout the mixing, injection, debinding, and sintering stages. Since all steps in the process are interrelated, it is important to have a general picture of the whole process which needs a scientific overview. This paper reviews the potential of LPIM and the characteristics of all steps. A complete academic and research background survey on the applications, challenges, and prospects has been indicated. It can be concluded that although many challenges of LPIM have been solved, it could be a proper solution to use this process and materials in developing new applications for technologies such as additive manufacturing and processing of sensitive alloys.

## 1. Introduction

Powder injection molding (PIM) is a manufacturing technology used to create small-to-medium-sized, highly complicated geometric components with remarkable dimensional and high precision at high production volumes [1,2,3,4]. This process comprises metal injection molding (MIM) and ceramic injection molding (CIM) [5] and uses metal or ceramic powder and polymer binder as raw materials. Binder systems are polymer-based multicomponent mixtures that play a crucial role in the manufacturing and defining of the PIM-produced products’ final qualities [6,7]. The PIM method offers the advantages of injection molding, including rapid manufacturing of complex shapes at a cheap cost and using powder metallurgy with superior mechanical qualities [8,9]. These characteristics have made PIM a competitive method for producing metallic and ceramic components compared to other precision processes. PIM comprises four main process steps: feedstock preparation (i.e., mixing), injection molding, debinding, and sintering [10,11]. In the first stage, the feedstock is produced by mixing the powder with a binder system at suitable and optimized proportions [12]. The feedstock is then heated and injected under pressure into a mold with the proper geometry to produce “green” components. Afterward, the green specimens are treated with various processes to remove all binder ingredients during the debinding phase [13,14,15]. The debound parts, called “brown” parts, need to be sintered to achieve high physical and mechanical properties values [16]. Based on the pressure used in the injection molding stage, PIM can be divided into high-pressure powder injection molding (HPIM) and low-pressure powder injection molding (LPIM) [17,18]. In HPIM, feedstocks with viscosities between 100 and 1000 Pa·s require high injection pressures between 20 and 200 MPa, which are given by the standard HPIM method [19,20].

In contrast, the LPIM method uses low-viscosity feedstocks to take advantage of injection pressures between 10 and 500 kPa (i.e., less than 1 MPa). LPIM has been continually developed over the past fifty years [20,21]. Additionally, this process is a desirable injection technique for micro-part production by micro-PIM because of its low equipment costs, simple mold filling control, and low maintenance costs [22]. In addition, the process’s energy consumption, the mold wear rate, and the relative absence of a density gradient in the molded part might be deemed to have significant benefits. However, lower injection pressures are only attainable when the injectable mixture contains a relatively high fraction of binders with low viscosity. Therefore, the use of LPIM has been hampered primarily by the problems associated with binder removal and powder-binder separation, especially for high-density powders. Additionally, later it is shown that segregation plays a vital role. Moreover, numerous crucial facets of LPIM are still poorly understood.

**Figure 1:**
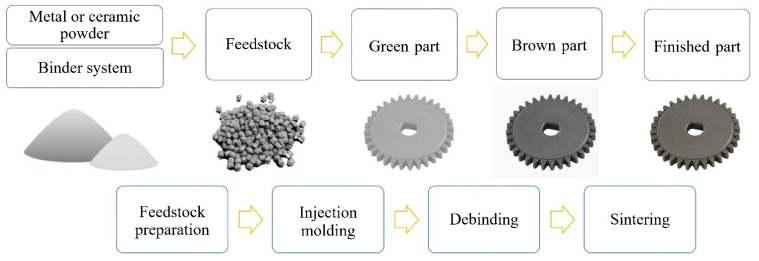
Material flow and processing sequence in LPIM process.

Recently, LPIM has been widely promoted as a technique for prototyping and small series production [23,24,25]. Initially used in ceramics forming [26], the LPIM technique has rapidly become acceptable for producing metallic components with high added value in aerospace and medical applications [27,28,29]. Research groups have used LPIM to produce parts out of metals, ceramics, and carbides, including 316-L stainless steel [30], Inconel 718 superalloy [31], alumina [32], cemented tungsten carbide [33], and near-net-shape ceramic parts in the range of 100–10,000 units [34]. Current research focuses on developing experimental methods and numerical simulations to examine the impact of powder characteristics, injection parameters, and feedstock features on metal powder and binder segregation during injection molding [35,36,37]. Recent developments in LPIM can be attributed to the impressive growth rate of conventional PIM technology and the new requirements brought in by the automotive, medical, and aerospace sectors [38]. The schematic view of the LPIM process is shown in Figure 1.

Even though this method presents several obstacles compared to HPIM, LPIM can be an effective process due to the reduced size of the injection machine, the elimination of multiple debinding procedures, and the substantial reduction in manufacturing costs [20]. In addition to the abovementioned benefits, the shear forces at the tool walls and cavities are decreased, which is particularly advantageous when employing extremely abrasive hard materials. This occurrence would prevent the harmful contamination of powders by other elements. Furthermore, following injection, the molded part has reduced internal pressures. A low viscosity multicomponent binder in LPIM has to ensure excellent molding capabilities and promotes powder-binder interactions and feedstock stability, resulting in little segregation. For a low viscosity, the binder system in LPIM often excludes polymers with a high molecular weight [22,39]. The powder loading cannot exceed a specific value which depends on powder characteristics, binder system, and processing parameters. Using higher powder loading for injection is not possible or might generate process-related issues and complications such as agglomeration, inhomogeneity in the feedstock and different defects in each step. Due to the absence of elements in the binder that help in shape retention at high temperatures, the debinding and sintering processes should be performed with extra care. In addition, it might be challenging to attain excellent mechanical characteristics with less powder loading compared to HPIM. In most instances, the solvent debinding is eliminated from the LPIM [40]. The green components are embedded in a wicking powder bed without or with a reduced backbone content, and the thermal debinding is carried out in a protective atmosphere. In addition, using a low viscosity binder during the injection molding step might result in various flaws, such as trapped air spaces. A comprehensive analysis of the LPIM characteristics and circumstances is required to overcome these challenges. In this paper, all aspects of the LPIM process, including preparing a suitable feedstock, injection molding, debinding, and the prospects, have been discussed profoundly for the first time by considering all metallic and ceramic materials used in LPIM, which different researchers have investigated.

Following this introduction, this review study is divided into 6 sections. Section 2 describes the ideal raw materials’ features, including the powder and binder systems. Section 3 introduces all techniques for characterizing feedstocks, including rheology, thermal analysis, and several methods for determining the homogeneity of feedstocks in LPIM. Section 4 explains the injection molding process, one of the most crucial steps vastly distinct from HPIM, in three subsections: injection machine and mold, parameters and defects, and simulation. Section 5 discusses the debinding procedure, which is essential owing to using waxes as a binder. Section 6 describes the sintering process that generates the finished product. Section 7 concludes with a summary of the review article, views for further development, and intriguing suggestions for future investigation.

## 2. Materials and Methods

The first step of the LPIM process is preparing feedstock materials consisting of metal or ceramic powders and a binder system consisting of various organic components. This section presents the ideal characteristics of the powders (Section 2.1) and the binder components (Section 2.2) to perform LPIM successfully. Examples of the powders successfully processed by LPIM are shown in Table 1, and the compositions of valuable binders in LPIM are shown in Table 2. This section aims to inform the reader of the features to consider when selecting powders and binder ingredients for LPIM feedstock materials since not all powders and binders might be processable by LPIM.

### 2.1. Powder

Metal and ceramic powders can be used for PIM if they have a small enough size (<45 μm). They are loaded into a polymeric carrier and can be sintered to a high enough density. Different techniques can be used to create these powders like gas atomization, water atomization thermal decomposition and chemical reduction [2]. Each method will influence the powder characteristics and, therefore, the final component’s density, dimensions, and distortion [37,41,42,43]. Suitable PIM powders are sufficiently small and sinterable but do not exhibit strong sintering behavior at temperatures where binders are removed. The ideal powder characteristics for this process can be mentioned as follows [2,44,45]. Most alloys should have particle sizes of less than 22 μm with high surface purity to maintain homogeneous interaction with polymers and enhance sintering. An ideal powder must have a spherical particle shape and a high packing density to load as much powder into the polymer as possible. Other required parameters are appropriate interparticle friction and void-free non-spongy particles, contributing to high final density and low explosivity and pyrophoricity to prevent accidents. The binder system greatly influences the mixing, injection, and debinding steps. However, powder characteristics such as particle size, particle size distribution (PSD), and shape also determine the feedstock processability and the final sintered properties [46,47,48,49]. Water and gas atomization are two standard methods for producing irregular and spherical powder particles, respectively. Feedstocks made from a gas-atomized powder have greater injection molding capability, although they are often more expensive than feedstocks formulated from a water-atomized powder. This improved moldability is attributed to a reduction in interparticle friction as well as a reduction in the energy required to align the powder with the flow during the injection. The significant distinction between water and gas-atomized particle manufacturing is that gas atomization makes spherical particles due to its smaller heat-carrying capacity. In contrast, water atomization, which has the largest heat-carrying capacity of any liquid, produces irregular shapes for the powder particles [50,51,52,53,54].

When controlling distortion and defects in PIM, especially in LPIM, choosing the right powder with a suitable particle size is one of the most important factors [55]. Predominantly small particles are preferred to allow better sinter densification of PIM components. Additionally, using smaller powder sizes significantly reduces surface roughness [56,57]. For example, You et al. [58] used a bimodal type powder mixture, including Fe micro-nano powder, which improved the surface roughness of the sintered surface. According to this research, this effect results from the nano powder filling the cavities among the micro powders. Smaller powders, conversely, cause agglomeration and make debinding more difficult since capillary channels between particles are smaller [59,60,61]. However, this reduced capillary channel size increases the binding between powder particles and the binder system [62]. Because of particle aggregation, achieving a high packing density with small powder particles could be challenging. Furthermore, as the particle size is smaller, getting a homogeneous feedstock requires more energy and time. Additionally, larger particles (>30 μm) can be employed if cost is a factor. However, larger particle size can cause process issues such as screw seizure due to the trapped particles between the check ring and the barrel and requires careful choice of tolerances for the check-ring [2]. In addition, the strength of brown parts is decreased, leading to a high probability of increased distortion and defect formation. A larger clearance check ring in the molding operation is preferable in the case of using larger particles. Additionally, when larger particles are pressed together, their intrinsic strength is diminished compared to smaller particles since larger particles have fewer particle-to-particle interactions per unit volume. On the other hand, when particle size reduces, feedstock viscosity increases, which in certain situations causes the process to fail, notably in the LPIM, which employs low pressures [63]. Furthermore, some reactive powders, such as titanium, cannot be made in sizes smaller than 1 µm due to their pyrophoric behavior [64]. Ghanbari et al. [65] investigated the impact of micro-sized and nano-sized SiC powder on the rheological behavior of Al-based feedstocks for LPIM. Micro-SiC has little effect on viscosity, but nano-SiC significantly increases viscosity. This was explained by the impact of SiC particle size on particle interactions. Gholami and Demers [66] compared two Co-Cr powders with similar powder sizes and distributions but different surface textures. It was shown that due to a more textured surface, the injection properties, e.g., injection length and moldability index, decreased.

The powder’s particle size distribution (PSD) is also a vital characteristic due to its effects on several factors, including surface area, rheology, and packing density. The d_10_, d_50_, and d_90_ numbers can be used to describe a PSD. The d_10_, d_50_, and d_90_ particle sizes indicate that 10%, 50%, and 90% of the particles are smaller than the specified number, respectively. The d_10_ and d_90_, in theory, represent particle sizes approaching the particle size distribution tails. Typically, powders are offered in d_90_ sizes; for instance, a d_90_ of 22 μm indicates that 90% of the particles are smaller than 22 μm. A smaller d_90_ means finer particles are present and, therefore, sinter better, but the loading into the binder system may be inadequate. Because there are fewer particle-to-particle interactions per unit volume as the particle size increases, a large d_90_ will have more coarse particles, sinter poorly, and may show deformation or cracking [2]. It is crucial to know the PSD to ensure that the feedstock produced during the PIM is consistent. Compounding, molding, and sintering can differ for two powders with the same d_50_ but varying d_90_ and d_10_ composition [67]. Another parameter to describe a PSD is the width of the distribution. It has been observed that a broader PSD could result in a denser final product and less binder needed in the feedstock during the process. S_W_ can be used to describe the width of a PSD, and Equation (1) is used to calculate S_W_ [16]:

(1) $$S_{W}=\frac{2.56}{\log(\frac{d_{90}}{d_{10}})}$$

A lower value of S_W_ indicates a more extensive particle size range, which facilitates injection throughout the process, whereas a greater value of S_W_ results in a low-density component. For example, powders with S_W_ values of 4 and 5 are difficult to inject, while powders with S_W_ higher than 7 are totally unsuitable for injection molding. [52,68]. Several studies have demonstrated that PSD can significantly affect properties like rheology [69,70]. However, Sotomayor et al. [52] proved that only some parameters, like viscosity and optimized powder loading, could be influenced to a large extent by PSD.

Furthermore, the shape of the powder has a significant impact on the process parameters and final quality. Spherical powders are recommended for the feedstock’s increased packing density and flow characteristics [71]. Nevertheless, the irregular powder form resulted in better dimensional precision when compared to spherical particles of the same mean size [16]. Due to the interlocking of irregular particles, this form promotes shape stability in the green and debound specimens [72]. Improved shape retention for spherical particles can be obtained by reducing particle size to get more outstanding particle-to-particle contacts per unit volume. The more spherical the powder, the higher the packing density, leading to higher powder loading used in the feedstock.

**Figure 2:**
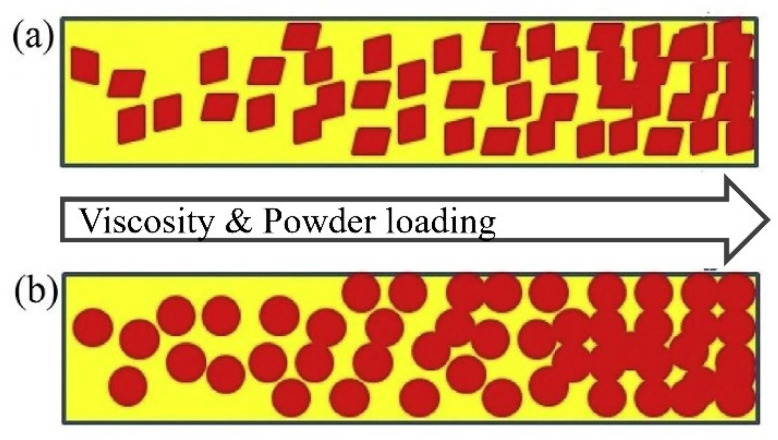
The influence of powder particle shape on viscosity: (**a**) non-spherical particles; (**b**) spherical particles. Reproduced with permission from reference [74], copyright © 2020, Elsevier B.V.

Consequently, spherical powders can potentially lower shrinkage than non-spherical powders. According to some literature, the main advantages of spherical morphology are reduced viscosity and good packing, which are favorable in the injection molding process and prevent the void formation in injected parts [42,58]. For example, Aslam et al. [42] stated that powder particle shapes also influence solid loading and result in viscosity differences, with spherical particles having a lower viscosity than disks, rods, and ellipsoids. Despite this, Loh et al. [73] used 316 L stainless steel and titanium carbide (TiC) powders during PIM, indicating that irregular particles retain molded sample forms with excellent quality during debinding. In general, when particle sphericity decreases and particle surface roughness increases, viscosity increases. As seen in Figure 2, in die cavity filling, the inability of non-spherical powder particles to rotate on one other inhibits their mobility. This causes non-spherical powder particles to have a greater viscosity than spherical powder particles [74]. Hidalgo et al. [75] verified that using spherical and small ceramic powders translates to better injection and sintering. You et al. [58] indicate that the activated sintering of Fe nano powders improves the sintering of Fe micro powders in Fe nano-micro powders at elevated temperatures, which results in stronger green and final parts.

Some research groups have focused on the effect of powder shape and size on rheological behavior in LPIM feedstocks, primarily for ceramic-based powder-binder mixtures. Schlechtriemen et al. [76] used two similar ceramic mixtures with different specific surfaces and morphology to tailor the powder characteristics providing suitable feedstock properties for the LPIM process.

In another study, the influence of particle size and particle shape on rheological, thermal, and segregation properties were investigated for typical 17-4 PH feedstocks used in the LPIM process by Trad et al. [36]. Four different powder lots were used in this study with the d_90_ of 5.4, 20.3, 31.3, and 27.5 μm, respectively. It was shown that the particle size and shape regulate the specific heat capacity. The shear-thinning behavior, generally suitable for the LPIM process, was observed over the main shear rate range for all feedstocks. Irrespective of the powder size or shape, this phenomenon is mainly driven by binder molecule orientation and ordering within the flow. Additionally, the S_W_ was 3.91, 2.7, 2.65, and 2.66, respectively. It has been demonstrated that feedstocks formulated with coarser and greater spherical particles have a higher moldability potential. The results also show that a coarser powder does not affect the molding qualities during the injection.

Additionally, Majdi et al. [49] used the LPIM approach to make iron parts using four different irregular particles (5.0 < d_50_ < 18.3 μm) and spherical (d_50_ = 4 µm) iron-powders and a standard binder system containing paraffin wax (PW), carnauba wax (CW), stearic acid (SA), and ethylene vinyl acetate (EVA). The four feedstocks were injected into a rectangular mold cavity. The feedstocks’ low viscosity and melting point confirmed that the LPIM process could shape irregular iron powders sieved at <45, <20, and <10 μm, as well as spherical iron powder with a d_50_ as low as 4 μm. Furthermore, the highest solid loadings observed with irregular (56–58 vol.%) and spherical (62 vol.%) powders represent the expected values for these two powder morphologies.

As shown in Table 1, several investigations in LPIM have been conducted for various metallic and ceramic powders. All metallic and ceramic powders successfully employed in LPIM to make high-quality parts are listed in Table 1. The most frequent material used in this challenging process is alumina (Al_2_O_3_) powder, while for metals are iron and steel. It is noticeable from the table that ceramic powders are frequently made up of smaller particles. This can result in increased viscosity, which will be discussed more in the binder system selection section. One important aspect when selecting the powder to be used in the feedstock is the dimensions of the specimen to be shaped. For example, if micro-parts or parts with microfeatures are to be shaped, the particles should be smaller than 1 µm for the cavity to be adequately filled [25]. However, LPIM is quite versatile, and it can also be used for parts with dimensions as large as 165 mm [49].

**Table 1:** Powders and characteristics of the powders used in the LPIM process.

Powders	Particle Size				Powder Loading		Green Part’s Largest and Smallest Dimensions (mm)	References
	µm		nm					
	d_50_	d_90_	d_50_	d_90_	Wt.%	Vol.%		
Al_2_O_3_	0.4				85		50, 3	[77]
	0.5, 5				87.5		50, 5	[78]
	0.4				85		49.95, 4	[79]
	2.6				80		N/A	[80]
	0.4				86		63, 10	[81]
	0.4				86		N/A	[26]
	0.4					50	60, 4	[82]
	0.4				86		26.5, 4	[83]
	1.3	1.9				65, 75	20, 0.074	[32]
	3					60	80, 10	[84]
	3					60	120, 3.5	[85]
	3					60	120, 3.5	[86]
	0.4				86		20, 3	[87]
	0.4				86		50, 1.5	[88]
	0.4				86		63, 10	[18]
	2				80, 82		40, 3.2	[89]
Al_2_O_3_ + ZrO_2_ + MgO	Al_2_O_3_ = 0.38, ZrO_2_ = 10, MgO = 1					55	N/A	[90]
Al + Al_2_O_3_ + ZrO_2_	1.48				84.1	55.5	45, 5	[91]
	1.70				86.4	60	45, 5	[91]
	1.96						45, 5	[91]
Zirconia	0.25					55, 57, 58, 60	N/A	[92]
	0.59				85		24, 2	[83]
			400			52	1.4, 0.19	[25]
Granite	-	-	-	-	82	62	<10, <10	[93]
Stainless steel (316L)	8.25	20.24				62	N/A	[94]
	-				93.16		N/A	[30]
	10				-	-	N/A	[95]
	6.9, 20					63–70	N/A	[29]
	6.7					60	N/A	[35]
Al + Al_2_O_3_ + ZrO_2_	1					56–66	N/A	[96]
Al + AlN + TiB_2_	Al = 24, AlN = 6.24, TiB_2_ = 20				82		N/A	[97]
High-speed steel (T15)	22				94.28		N/A	[98]
ZrSi_2_ + ZrO_2_			ZrSi_2_ = 183, ZrO_2_ = 63			57, 60	15, 0.1	[99]
ZrSi_2_ + ZrO_2_ + Al_2_O_3_ + MgO			ZrSi_2_ = 183, ZrO_2_ = 63 Al_2_O_3_ = 12, MgO = 67			57, 60	15, 01	[76,99]
Al_2_O_3_ + steatite	Al_2_O_3_ = 1.9, Steatite = 4.8	Al_2_O_3_ = 4.2 Steatite = 9.5			87.8, 88		60, 10	[100]
	Al_2_O_3_ = 1.9, Steatite = 4.8	Al_2_O_3_ = 4.2 Steatite = 9.5			88		60, 3.5	[40]
Iron	4.1		100			66	10, 5	[58]
	16.6	44.1				55	165, 3	[101]
	16.6	44.1				50–68	N/A	[102]
	18.3	44.9				58	165, 3	[49]
	6.5	19				58	165, 3	[49]
	5	14.1				56	165, 3	[49]
	4	7.7				62	165, 3	[49]
Zirconium silicate	1.6	4.65				57.5	60, 4	[103]
Nickel oxide (NiO)-yttria-stabilized zirconia (YSZ)	-	-	-	-	-	-	27, 1	[104]
Cemented tungsten carbide	0.67					50	9, 0.9	[33]
Inconel 718 superalloy	12					60	N/A	[31]
	12					60	N/A	[105]
	12					60	35, 9.5	[106]
	12					60	35, 9.5	[107]
	12					50, 60	N/A	[108]
Inconel 625	12.5	27.8					N/A	[38]
Al + SiC	Al = 28		SiC = 40		78, 79, 80, 81		N/A	[41]
	Al = 20, SiC = 28		SiC = 40		80		N/A	[65]
B4C	14					55	N/A	[109]
Co-Cr	-	-	-	-		50–70	N/A	[66]
NiO-YSZ	-	-	-	-		-	27, 1	[110]
Stainless steel 17-4PH	2.7					60	35, 9.5	[36]
	7					60	35, 9.5	[36]
	11.8					60	35, 9.5	[36]
	11.3					60	35, 9.5	[36]
	12					60	100, 11.75	[37]
	11.8	31.3				60	165, 3	[111]
SiC	15					65.12, 64.70	55, 6	[112]
	0.1						N/A	[20]
	3, 15					60	55, 6	[113]
Si-Y_2_O_3_-Al_2_O_3_-MgO	0.7–1.8					55.6–61	20, 2	[23,24]
Magnesium aluminate (spinel)				100	75–85		120.1, 3.5	[114]
Ti	29	54				53, 60, 65	38.5, 1.5	[74]
Ti-6Al-4V	18					60–72	76, 2.5	[115]
	14.9	23.9				63	60, 6	[116]
Y_2_O_3_ + Al_2_O_3_ + CeO_2_	0.39				70–78		100, 2	[117]

Powder selection is crucial for the success of the LPIM process. The powder’s particle size distribution and shape significantly influence the flowability of the feedstock, which influences the homogeneous filling of the cavity features. Thankfully, much research has been done, and Table 1 can be used to select metal and ceramic powders with a suitable particle size distribution that can successfully be used in LPIM. However, not only the powder characteristics influence the behavior of LPIM feedstock, but also the binder system; Therefore, it is essential to describe the binder system in detail in the next section.

### 2.2. Binder System

Binder systems are polymer mixtures with several components. Binders help retain the molded shape and keep the powder particles together until the sintering begins. The binders are combined with powders to create feedstocks, which are then used for injection molding. The properties of binder systems affect the dispersion of particles, the shaped component’s dimensions, the shaping process, and the sintered component’s final quality. For this reason, the binder system is essential in PIM and even more critical in LPIM. A binder comprises a primary ingredient and additional additives such as dispersants, stabilizers, and plasticizers [2]. During debinding and the initial stages of sintering, the backbone helps to stabilize the shape of the injected feedstock. The backbone is also used as a thickening agent to increase the mixture’s viscosity and help surfactant to prevent powder-binder separation. The use of dispersants improves powder particle dispersion and increases powder loading. During injection molding, plasticizers are utilized to enhance the flow of the feedstock. Low molecular weight polymers are added to the backbone polymer as lubricants to minimize friction and viscosity between powder particles. Surfactants help disperse the powder within the polymer matrix and prevent agglomeration [51,118]. The different polarities of powder and binder make it difficult for them to adsorb each other, and surfactants are usually applied as a connecting medium. The carboxyl group of surfactants is polar, which can combine powder, whereas the alkyl group is nonpolar, combining the binder, according to the ‘like dissolves like’ concept [110].

There are some prerequisites for an ideal binder system. Generally, the binder system should have good adhesion to the powder, low contact angle, chemically passive, low viscosity, and low residual carbon content after removing from the part. Although most of the requirements of the binder system for LPIM are the same as HPIM, it is more challenging due to the low pressures to inject the feedstocks. Particles should have a low contact angle with the binder. Because of the low contact angle, the binder will be better wetted to the powder surface, aiding the mixing and molding process. The binder and filler particles should be inert. The binder must meet specific rheological parameters for appropriately molding the components without producing flaws. Powders and binders will separate if the binder’s viscosity is too low during processing. A high binder’s viscosity, on the other hand, will impede the mixing and molding process. Because of low temperatures and pressures in LPIM, PW or low-viscosity waxes with low melting temperatures should be used instead of standard binder systems for HPIM. Binder systems used in LPIM can be seen in Table 2. It is shown that PW, CW, and BW are the most common waxes to use in LPIM. Considering the type of PW, it is more suitable to use low molecular weight PW grades with low melting temperature (45–65 °C) and essentially low viscosity. PW, as a soft thermoplastic polymer consisting of hydrocarbons, is the most extensively used wax in LPIM binder systems in which the content can sometimes reach 90 vol.% of the binder system [119]. It is notable from Table 2 that waxes have usually been used as the main components of binder systems for ceramic feedstocks. However, it is possible and helpful to use a small proportion of backbone in the binder system for metallic powders. Generally, finer powders have been used for ceramics, making it more challenging to use backbones in most cases of this process. CW, a natural wax formed on Brazilian palm leaves, can play an essential role in developing proper binder systems for LPIM due to its great potential in particle wetting, gradual division of the debinding process, and lubrication [5,120]. According to the melting temperature, degradation behavior, and viscosity, CW could be a reliable alternative for the backbone in LPIM because it helps to retain the shape during thermal debinding. Compared to PW, CW is a polar wax that can improve the interaction between binder constituents and powder particles, resulting in better homogeneity and processability of the feedstock. The applicability of CW as a backbone has been recently demonstrated [121]. In the case of using non-wax polymers as the backbones in LPIM feedstocks, polyolefins such as low viscosity grades of PP, PE, and EVA are usually used. When it comes to using polyolefins as the backbone, although some researchers have used high percentages of polymers successfully [33,74,79], it is recommended to have less than 10 vol.% polymers in the binder system for LPIM to keep the viscosity low.

The surfactant is another component essential for binder systems used in LPIM [122]. The surfactant could create a thin surface layer between powder particles and binder constituents. This layer allows particles to slide, which results in reducing the viscosity of the feedstock [123]. SA and oleic acid (OA) are predominantly employed as surfactants in LPIM. At least one can be found in every binder system, demonstrating the significance of these two surface-active ingredients. By having a functional group clinging to the powder surface and an oriented molecular chain extending into the binder, SA avoids powder aggregation and stabilizes particles under severe shear [124]. According to Medvedovski and Peltsman [125], OA, with a melting temperature of 15 °C, is more effective in industrial LPIM. Some studies also reported problems like bubbles developing in the ceramic feedstock due to stearic acid evaporation or a high amount of residual carbon by using SA [26]. However, the result of comparing SA and OA by Tseng [123] showed that using SA exhibited superior pseudoplastic flow behavior than OA, and the viscosity could be reduced more effectively. According to some new binder systems for LPIM, using both SA and OA could be a suitable option to provide both advantages [18,26,81,88].

Generally, low viscosity feedstocks produced with low molecular weight polymers have been employed in LPIM for the past 15 years to improve moldability and shape complexity. Reduced feedstock viscosity results in higher moldability at lower pressure, reducing tooling costs and increasing defect-free components by preventing mold deformations [66]. Since LPIM feedstocks do not contain high molecular weight backbone polymers in many cases, their viscosity is determined by the waxes and short-chain additives. Medvedovski and Peltsman [125] state that PW is the preferred ingredient since it provides melting and low viscosity at low temperatures. Low molecular weight PWs are preferred when using PW for the LPIM. For some binder systems, small amounts of other waxes, such as BW, CW, and PE wax, can be used as plasticizing agents and surfactants since they have a good affinity for PW and help to reduce the viscosity even further. As previously mentioned, CW was designed to aid particle wetting and debinding. In summary and as seen from Table 2, different waxes are used as primary components in most cases, mainly when mixed with ceramic powders. A small content of polymers such as PE, PP, LDPE, EVA, and HDPE as a backbone could be used mainly for metallic powders.

A partially water-soluble binder system for the LPIM of alumina powder has successfully been developed and tested by Bakan and Gunes [82]. They designed a binder system consisting of Poly (2-ethyl-2-oxaline) as the water-soluble portion, LDPE for high strength during debinding, SA, and a little concentration of a gelling agent to decrease the viscosity. LDPE is a thermoplastic polymer used as the backbone in feedstock preparation for micro-PIM and LPIM. For example, Kong et al. [126] have used the LDPE to develop a 316 L stainless steel feedstock to fabricate micro-components, with a powder loading equal to 66 vol.%. Using LDPE helps to obtain good homogeneity, the most negligible contamination, and suitable fluidity, as reported [126]. The major components of the binder, i.e., (Poly (2-ethyl-2-oxaline), can easily be removed in a relatively short time by water leaching at about 60 °C for six hours. It opens pore channels that allow much faster removal of the remaining binder during subsequent thermal debinding. Bakan and Gunes confirmed that higher heating rates than those used for conventional binder systems could be employed for thermal debinding and sintering procedures when employing this binder system for alumina injection molding, which reduces the total cycle time of the process.

In the research work from Trad et al. [36], the solid loading was set at 60 vol.% powder to make four different feedstocks with the same low melting point binder system of 30 vol.% PW, 7 vol.% CW, 2 vol.% SA, and 1 vol.% EVA. PW and CW were selected to help with part ejection after mold filling, SA was used to promote the surfactant effect that strengthens chemical bonds between powder and binder, and EVA to produce a thickening effect necessary to control powder segregation. According to Demers et al. [31], adding 5–10 vol.% of EVA in PW results in a considerable thickening effect. The minor binder was composed of LDPE and EVA. Compared to LDPE, the polar EVA side group increases the force between molecules, enhancing adhesion and form retention after binder removal. Ghammi and Demers [116] note that a titanium-based feedstock containing only waxes and surfactant agents requires the addition of a thickening agent (i.e., EVA) due to the low viscosity and possible powder separation. Fareh et al. [105] examined the effect of low viscosity binder systems on the thermal and rheological properties of several Inconel 718 superalloy feedstocks. They confirmed that PW, bee wax (BW), and SA could decrease the viscosity of the Inconel 718 feedstock at different temperatures, and EVA increases the viscosity. They also observed that a mixture without a polymer backbone and surfactant agent (i.e., 35PW-5EVA) shows near-Newtonian behavior. The results also showed that the binder systems containing SA and PW could be suitable options.

Côté et al. [115] investigated the effect of binder ingredients and solid loadings on the rheological behavior and moldability of titanium-based spherical powder feedstocks utilized in an LPIM process. Thirty-five different feedstocks were formulated with one spherical plasma-atomized titanium powder (d_50_ = 18 μm) and four wax-based binder constituents using powder loadings varying from 60 to 72 vol.%. PW, SA, EVA, and CW were used as the primary carrier, surfactant, thickening agent, and anti-shrinking agent, respectively. During the development phases, viscosity profiles were utilized to identify the threshold proportions of each binder ingredient to maximize moldability while minimizing segregation. When SA is used as a surfactant agent, adding 1 vol.% of it to titanium-based feedstock is enough to decrease the feedstock viscosity without significantly affecting the thermal sensibility. When EVA is used as a thickening agent, a minimum proportion of roughly 2 vol.% of it is required to reduce the segregation for a powder loading of 66 vol.%. However, this proportion of EVA significantly decreases the feedstock moldability and cannot be used to fill a complex shape mold cavity. When CW is used as a shrinking agent, a proportion of about 3 vol.% of it represents a good tradeoff between the feedstock moldability and the part demolding. In this study, the best candidate feedstock to produce complex shape parts is a mixture containing 64, 1, 1, and 3 vol.% of titanium powder, SA, EVA, and CW, respectively. This binder formulation was also used to produce feedstocks exhibiting high solid loading varying between 66 and 68 vol.% of powder.

In addition, the impact of binders on the viscosity of LPIM Inconel 718 superalloy was investigated by Demers et al. [31]. This study used CW, BW, EVA, SA, three grades of PW (i.e., PW1, PW2, and PW3), and two grades of microcrystalline wax (MW) in different binder systems. According to the findings of this study, PW1, BW, and SA may be used to reduce the viscosity of the feedstock, whereas PW2 and PW3, CW, and EVA are intended to enhance the viscosity of the feedstock. Most of the polymer constituents demonstrated rheological and thermal interactions when blended. The solubility of polymers is generally preferred to finely disperse the constituents, homogenize the polymer blend, and tailor the mixture’s rheological properties. When they are used to prevent powder-binder separation, the solubility of thickening agents in wax-based binders is also desirable to minimize their impact on injection temperature. It was also shown that 5–10 vol.% of EVA must be added in PW to observe a significant thickening effect and that the PW2 and PW3 can be potentially added to low viscosity PW in order to play a similar role to that of EVA at low shear rate. Adding only 1 vol.% of SA in PW generates a significant decrease in viscosity, but further increasing in SA proportion does not improve the rheological behavior of the feedstock. From the rheology perspective, there is no significant advantage in using MW or CW as a central component of the feedstock. The best candidate feedstocks are the mixtures containing PW1 and SA, while feedstocks based on PW1, BW, or including a small amount of EVA could also be considered acceptable. Therefore, these constituents can be used as the main waxes for optimizing more complex LPIM feedstock in future works.

Finally, it may be concluded that from a rheology point of view, it is good to design a binder system without a backbone polymer for LPIM. However, as it could be realized from the literature, having a small amount of backbone improves the results and eliminates the injection and debinding defects. When deciding to use a backbone, it is better to consider polymers with very low viscosity and melting point grades.

Backbone could be useful if the entire debinding process is done in stages because, without any backbone, the debinding must be carried out only thermally. Even in this procedure, a backbone can prevent a complete loss of shape during the part softening.

**Table 2:** Current examples of binder systems and their respective powders used in the LPIM process.

Powders	Binder Components	Green Part’s Largest and Smallest Dimensions (mm)	References
Al_2_O_3_	PW, branched PW, dispersing agent	50, 3	[77]
	PW, SA	50, 5	[78]
	70% PW, 25% PP, 5% SA (wt.%)	49.95, 4	[79]
	CW, LDPE, SA	N/A	[80]
	75% PW, PE wax, CW, SA, OA (wt.%)	63, 10	[81]
	75% PW, PE, CW, SA, OA (wt.%)	N/A	[26]
	P2E2O, LDPE, SA	60, 4	[82]
	91% PW, 6% BW, 3% OA (vol.%)	80, 10	[84]
	91% PW, 6% BW, 3% OA (vol.%)	120, 3.5	[85]
	91% PW, 6% BW, 3% OA (vol.%)	120, 3.5	[86]
	75% PW, 10% MW, 10% CW, 3% OA, 2% SA (vol.%)	50, 1.5	[88]
	75% PW, 10% PE, 10% CW, 5% SA and OA (vol.%)	63, 10	[18]
	80.56% PW,19.44% SA (wt.%) 80.56% BW, 19.44% SA (wt.%) 50% PW, 35% BW, 15% SA (wt.%)	40, 3.2	[89]
Al_2_O_3_ + ZrO_2_ + MgO	88.9% PW, 6.7% OA, 4.4 PE (vol.%)	N/A	[90]
Al + Al_2_O_3_ + ZrO_2_	64.15% PW, 13.84% SW, 13.84% ODA, 8.17 MFO (wt.%) 69.12% PW, 15.44% SW, 15.44% ODA (wt.%)	45, 5	[91]
Granite	94% CW, 5% LDPE, 1% SA (wt.%)	<10, <10	[93]
Stainless steel (316 L)	59.2% PW, 14.5% PE, 21% CW, 5.3% MW (vol.%)	N/A	[94]
	55.3% PW, 23.7% CW, 20% EVA, 1% SA (wt.%)	N/A	[30]
	70% PW, 25% PP, 5% SA (vol.%)	N/A	[95]
	65% PW, 30% PP, 5% SA (vol.%) 70% PW, 25% PP, 5% SA (vol.%)	N/A	[29]
	100% PW, EVA, SA (vol.%) 75% PW, 12.5% EVA, 12.5% SA (vol.%) 75% PW, 25% EVA (vol.%)	N/A	[35]
Al + Al_2_O_3_ + ZrO_2_	PW, ODA, LOP	N/A	[96]
Al + AlN + TiB_2_	79% PW, 10% LDPE, 10% EVA, 1% SA (wt.%)	N/A	[97]
High-speed steel (T15)	60% PE wax, 35% PW, 5% SA (vol.%)	N/A	[98]
Al_2_O_3_ + steatite	98% PW, 2% SA (wt.%)	60, 10	[100]
	98% PW, 2% SA (wt.%)	60, 3.5	[40]
Iron	75% PW, 25% SA (vol.%)	10, 5	[58]
	93.3% PW, 4.4% SA, 2.2% EVA (vol.%)	165, 3	[101]
	PW, BW, SA, CW, EVA	N/A	[102]
	PW, EVA, CW, SA	35, 9.5	[36]
Zirconium silicate	30% CAB 381-0.1, 70% PEG 20,000 (vol.%) 30% CAB 551-0.01, 70% PEG 20,000 (vol.%) 30% CAB 553-0.4, 70% PEG 20,000 (vol.%) 30% LDPE 780E, 70% PEG 20,000 (vol.%)	60, 4	[103]
Cemented tungsten carbide	65% PW, 30% LDPE, 5% SA (wt.%)	9, 0.9	[33]
Inconel 718 superalloy	100% PW (vol.%) 100% MW (vol.%) 35% PW, 65% MW (vol.%) 100% BW (vol.%) 100% CW (vol.%) 50% BW, 50% CW (vol.%) 35% PW, 32.5% BW, 32.5% CW (vol.%) 97.5% PW, 2.5% SA (vol.%) 87.5% PW, 12.5% SA (vol.%) 75% PW, 25% SA (vol.%) 50% PW, 50% SA (vol.%) 97.5% PW, 2.5% EVA (vol.%) 87.5% PW, 12.5% EVA (vol.%) 75% PW, 25% EVA (vol.%)	N/A	[31]
	100% PW (vol.%) 100% BW (vol.%) 87.5% PW, 12.5% SA (vol.%) 87.5% PW, 12.5% EVA (vol.%)	N/A	[105]
	100% PW (vol.%) 100% CW (vol.%) 100% BW (vol.%) 50% CW, 50% BW (vol.%) 87.5% PW, 12.5% SA (vol.%) 50% PW, 50% SA (vol.%) 87.5% PW, 12.5% EVA (vol.%) 75% PW, 25% EVA (vol.%)	35, 9.5	[106]
	100% PW (vol.%) 97.5% PW, 2.5% SA (vol.%) 87.5% PW, 12.5% SA (vol.%) 50% PW, 50% SA (vol.%) 97.5% PW, 2.5% EVA (vol.%) 87.5% PW, 12.5% EVA (vol.%) 75% PW, 25% EVA (vol.%) 71.43% PW, 28.57% EVA (vol.%) 66.7% PW, 33.3% EVA (vol.%) 64.3% PW, 35.7% EVA (vol.%) 61.54% PW, 38.46% EVA (vol.%)	35, 9.5	[107]
	100% PW (vol.%) 87.5% PW, 12.5% EVA (vol.%) 87.5% PW, 12.5% SA (vol.%)	N/A	[108]
Al + SiC	PW, BW, SA	N/A	[41]
	89% PW, 9% BW, 2% SA (wt.%)	N/A	[65]
B_4_C	PW, CW, PP, SA	N/A	[109]
NiO-YSZ	PW, SA	27, 1	[110]
Stainless steel 17-4PH	75% PW, 17.5% CW, 5% SA, 2.5% EVA (vol.%)	35, 9.5	[36]
	75% PW, 17.5% CW, 5% SA, 2.5% EVA (vol.%)	100, 11.75	[37]
	75% PW, 17.5% CW, 5% SA, 2.5% EVA (vol.%)	165, 3	[111]
SiC	93% PW, 5% BW, 2% OA (wt.%)	55, 6	[112]
	PW, Surfactants	N/A	[20]
	PW, BW, SA	55, 6	[113]
magnesium aluminate (spinel)	PW, BW, OA	120.1, 3.5	[114]
Ti	69% PW, 27% LDPE, 4% SA (wt.%)	38.5, 1.5	[74]
Ti-6Al-4V	64.70–97.5% PW, 0–14.7% SA, 0–29.41% EVA, 0–29.41% CW	76, 2.5	[115]
	89.2% PW, 8.1% EVA, 2.7% SA (vol.%) 78.4% PW, 8.1% EVA, 13.5% SA (vol.%) 64.9% PW, 8.1% EVA, 27% SA (vol.%) 81.1% PW, 16.2% EVA, 2.7% SA (vol.%)	60, 6	[116]
Y_2_O_3_ + Al_2_O_3_ + CeO_2_	97% PW, 3% OA (wt.%)	100, 2	[117]
Si-Y_2_O_3_-Al_2_O_3_-MgO	PW, dispersants	20, 2	[23,24]
Zirconia	PW, dispersants	1.4, 0.19	[25]

Binder development is a complicated and time-consuming task usually done in a trial-and-error manner due to its multi-component nature. The LPIM binders are composed of multiple ingredients because of the numerous tasks the binder must accomplish. The binder must make the powder flow, prevent powder agglomeration, prevent phase separation, and decompose gradually, and it should not chemically react with the powder. As with the powder selection, numerous researchers have proposed several binder compositions that can be used with various metal and ceramic powders in the LPIM process. Therefore, it is recommended to use the examples shown in Table 1 and Table 2 in parallel to prepare LPIM feedstocks because, ultimately, it is the proper combination of binder and powder that ensures good quality parts obtained by LPIM. The following section describes the characteristics of feedstocks that should be measured to ensure that suitable feedstocks are used.

## 3. Feedstock Characterization

With a few significant exceptions, PIM feedstocks are molded similarly to polymers. However, these materials differ significantly from plastics in terms of thermal properties. The density is substantially higher than the binder components and is much closer to metal. This high density is because, while the amount of binder is nearly 40% in volume, the weight-based composition exceeds 90% when using metals. Analyzing the feedstock even more thoroughly is crucial for LPIM since the binder systems in this process are more sensitive to temperature or minor changes in the properties of materials than the binders for HPIM. The effectiveness of the LPIM is dependent on the characteristics of the feedstock, and this section aims to describe the experimental parameters that are crucial for determining the suitability of a feedstock for the LPIM process. The fundamental feedstock characterization approaches include a rheological analysis (Section 3.1) and an evaluation of its thermal properties (Section 3.2). Many parameters affect the homogeneity of the feedstock, which influence the mechanical and physical properties of the final parts [127,128]; therefore, it is necessary to determine the rheological and thermal parameters that are sensitive to feedstock quality, and this is described in detail in this section.

### 3.1. Rheological Properties

Melt rheology, or the flow characteristics of LPIM materials, is the most critical aspect of the feedstocks in this process [107]. The rheological properties of feedstocks significantly affect the success or failure of mold-filling. Improper rheological behavior may result in defects such as short shot, flash, cracking, bubbling, warpage, and powder-binder separation in the molding process [129]. The measurement of viscosity is performed with a capillary or rotational rheometer.

The rheological behavior of the feedstocks is complex due to temperature and shear rate dependencies [129,130]. It is reported and well-accepted that by increasing the volume fraction of powder in binder-powder systems, the viscosity of a feedstock will increase. Several semi-empirical models were developed to estimate feedstock’s viscosity dependency on solid loading. One of the well-known models is presented by Quemada:

(2) $$\eta =\eta_{b}{\left(1-\frac{\varphi }{\varphi_{\max}}\right)}^{-2}$$

where η_b_ is the binder viscosity, φ is the powder loading, and φ_max_ is the maximum powder loading in the system. Furthermore, it is reported that the viscosity of a feedstock decreases with an increase in the particle size and the particle’s sphericity. By designing feedstock formulations properly, a homogenous feedstock will show smooth and stable viscosity values over time. Depending on the material’s properties, viscosity changes may occur with increasing shear rates. The three classical rheological material behaviors are Newtonian, pseudoplastic, and dilatant. For the LPIM process, only shear-thinning (pseudoplastic) is favorable [131]. Shear-thinning facilitates cavity filling and enhances the shape retention of the green part. Here, the viscosity decreases with increasing shear rate. This behavior is due to the orientation and ordering of particles and the binder within the flow. The deagglomeration of the particles and formation of flow layers promote the decrease of shear rate and better dispersion and distribution of particles in the feedstock [31].

In comparison, the viscosity increases with increasing shear rate in dilatant (shear-thickening) material behavior [131]. Therefore, a suitable feedstock should not have a dilatant behavior since this can lead to an unfilled mold cavity. Equation (3), the Herschel–Bulkley model commonly cited in the literature, describes the flow characteristics of feedstocks. This model demonstrates the shear rate’s relationship with viscosity and can be used to investigate the effect of binder on feedstock rheological behavior in LPIM [103].

(3) $$\eta =k\cdot {\dot{\gamma }}^{n-1}+\tau_{y}$$

where η is the feedstock viscosity, $\dot{\gamma }$ is the shear rate, k is the consistency constant, n is the shear sensitivity index, and τ_y_ is the yield stress. Newtonian behavior of feedstock is recognized by n = 1. If n < 1, the feedstock behavior is pseudoplastic; if n > 1, the behavior is dilatant. Furthermore, the value of n specifies the behavior of the feedstock and its appropriateness for the injection step. Due to the feedstock’s high degree of shear sensitivity, a lower value of n is ideal for producing complicated geometry components. As a result, higher values of n indicate higher feedstock stability and improve shape retention [132]. A very low value for n initiates defects in molded parts, mainly due to the slip flow phenomena in the mixture [114].

In some works, it is reported that at high shear rates (in the range of the shear rates of injection molding), the feedstock shows dilatant behavior due to the segregation of powder and binder [114]. LPIM has been simulated by Moldflow (Autodesk, Melbourne, Australia). The choice of one or the other rheological models in the MoldFlow simulation depends on the viscosity profile. The Cross-WLF model is widely used when the viscosity remains constant at low shear rates and shows a significant decrease at medium and high shear rates. On the other hand, the second-order model is used when the viscosity quickly decreases at low shear rates and shows a Newtonian plateau at high shear rates (Equation (4)).

(4) $$\mathrm{Ln}\eta =A+B\mathrm{Ln}\dot{\gamma }+\mathrm{CT}+D{\left(\mathrm{Ln}\dot{\gamma }\right)}^{2}+E\mathrm{Ln}\dot{\gamma }+FT^{2}$$

where A, B, C, D, E, and F are the fitting coefficients. Another factor that has a massive influence on the viscosity of feedstock is temperature. For organic binders, the sensitivity of thermal changes is high [116]. The temperature dependency can be much more complicated in multi-component mixtures like binder systems [107]. Adding ceramic or metal particles to a binder will diminish the sensitivity, and injection molding can be done within a broader range of temperatures [29].

The relationship between temperature and viscosity has also been extensively researched [13,114,133,134,135,136,137]. Equation (5), the Arrhenius equation, which states that the viscosity of the feedstock decreases as temperature rises, can be used to explain this temperature dependency of viscosity.

(5) η=η0exp(ERT)

where $\eta_{0}$ is the reference viscosity, E the activation energy for flow, R is the universal gas constant, and T the temperature. The value of E reflects the degree of viscosity sensitivity to temperature change. By plotting lnη versus 1/T, one can calculate E by the slope. Therefore, the actual dependence of a feedstock’s viscosity on temperature can be derived by E. A high value of E indicates a high dependency of viscosity on temperature. Consequently, this results in a high tendency of feedstock to rapid solidification during injection molding [29]. A low value of E is suitable to minimize sudden viscosity changes between the injection press’s hot and cold zones. Furthermore, the E value decreases as the particle size increases [37].

The weir model (Equation (6)) is proposed for polymers and semi-solid materials to choose the most suitable powder loading in LPIM by rheological results. Calculating the moldability index (α), one can estimate the appropriate amount of solid content [79]. Moldability refers to a feedstock’s ability to be injected into a mold cavity without substantial flaws, and it is crucial for producing correctly complicated shapes or tiny components [138]. Because of the low viscosity in LPIM binder systems, injection defects could be formed easily [33,129,130]. The feedstock moldability α_STV_ can be described with the following Equation (6). As can be seen from the equation, the best feedstock corresponds to the highest value of α which is obtained for the lowest value of η_0_, n, and E, concurrently.

(6) $$\alpha_{\mathrm{STV}}=\frac{1}{\eta_{0}}(\frac{\frac{\partial \mathrm{Log}\eta }{\partial \mathrm{Log}\dot{\gamma }}}{\frac{\partial \mathrm{Log}\eta }{\partial (\frac{1}{T})}})$$

From the moldability perspective, a higher α_STV_ is desirable and means an easier filling stage in the injection molding. There is no exact range for α_STV_ in the literature since many parameters can affect the final value of the moldability index. It can be seen in the literature that researchers have found a wide range of α_STV_ between 9000 and 300,000 for suitable feedstocks [36,105,116]. Some investigations have been done on the rheological properties of LPIM. For example, the rheological behavior of Al-based feedstocks with micro and nano-SiC was studied by Ghanbari et al. [65]. The effect of the particle size on the viscosity and the activation energy was evaluated. Micro-SiC has little influence on viscosity; however, nano-SiC dramatically increases viscosity, according to the findings. The impact of SiC particle size on particle interactions explained this. In all micro and nano-feedstocks, the Arrhenius equation correctly describes the temperature dependence of the feedstocks. Furthermore, the activation energy was discovered to depend on the additional SiC particle size and shape.

In another work, Aslam et al. [29] studied the rheological behavior of LPIM of stainless steel feedstocks. A correlation between temperature and viscosity is established in this research, and the impact of a wax-based binder system on the flow behavior index and thermal properties for various feedstocks is evaluated. Different gas and water-atomized powder loadings, as well as different volume percentages of the binder components, were investigated. At 160, 170, and 180 °C, the flow behavior index values for all formulations are less than one, indicating that the feedstock has shear thinning behavior and that these formulations are suitable for LPIM. The activation energy E was determined for the different feedstocks. Their study confirmed that a powder loading of 62.76 vol.% stainless steel with 2.23 vol.% nano-sized boron and a binder containing PP, PW, and SA components have the lowest activation energy of 17.119 kJ/mol, making all these formulations suitable for LPIM at 170 °C. As mentioned before, Demers et al. [31] confirmed that using MW, or CW, as the major component of the feedstock has no substantial benefits. They considered minimum injection temperature and the rheological properties at different shear rates as a criterion to find the optimum binder system. Mixtures containing PW and SA are the best potential feedstocks; however, feedstocks based on PW, BW, or having a tiny amount of EVA may also be suitable.

### 3.2. Thermal Characterization

Thermal analysis can offer details on the thermal behavior of LPIM feedstock that could be helpful for the next steps. Differential scanning calorimetry (DSC) is utilized to monitor the melting and solidification temperatures of LPIM feedstocks. The DSC data can help to evaluate the ideal injection temperature for LPIM. Another technique is the thermogravimetric analyzer (TGA) which can be employed to assess particle segregation during the LPIM process. During the TGA analysis, binders are degraded, and the weight fraction of the residual powder can be calculated and converted to the volume fraction from the TGA profiles. The powder volume concentration data can predict the inhomogeneous distribution of the binder system in the feedstock. It should be done for samples of different sections in a green part by measuring the powder content. The powder content should be similar in all areas if no segregation occurs. This segregation phenomenon must be avoided to reduce distortions, warpage, fractures, and heterogeneous shrinkage during sintering.

**Figure 3:**
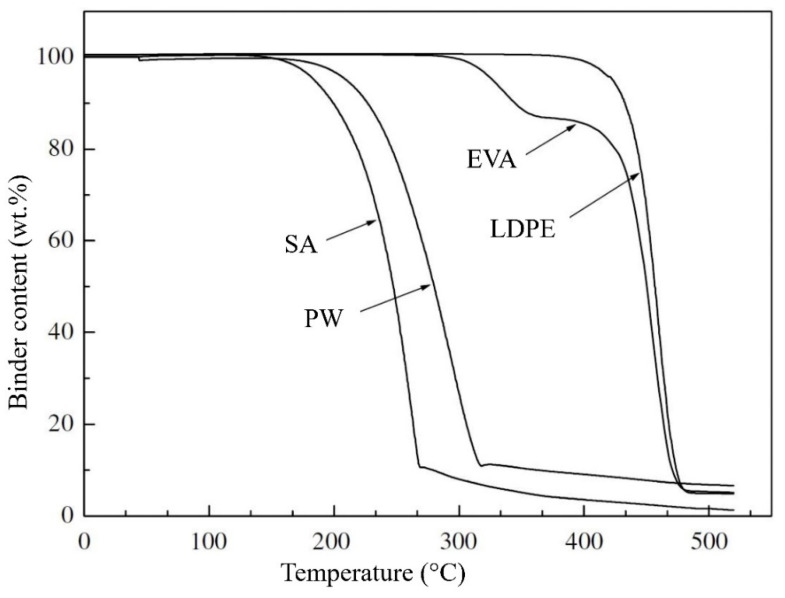
TGA curves of all binder constituents. Reproduced with permission from reference [97], copyright © 2004, Elsevier Ltd.

TGA data can also be used to investigate the decomposition of the binder system and, therefore, set the debinding temperatures. Figure 3 by Zhang et al. [97] depicts the weight loss characteristic of a binder system. The TGA curve is split into multiple stages according to temperature, depending on the binder component. The EVA curve shows two phases, with acetic acid being the only product of the first period before 360 °C and a weight loss of roughly 15%. In the next stage, it experiences a severe incline. Because of the low volatile temperature for short molecular chains, PW and SA degradation temperatures are lower than the rest of the components, and the slope gradients of these two components are minor.

Demers et al. [106] investigated TGA, pycnometer density (PD), and DSC for powder segregation measurement, concluding that these techniques can be utilized to determine the change in solid loading induced by segregation in LPIM feedstock. For the DSC, increasing the powder loading decreases the feedstock’s specific heat because the specific heat of the metal is less than the binder system. The enthalpy of fusion can be measured by using the area under the DSC peaks since it changes according to the specific heat of the feedstock. Therefore, it could be concluded that increasing the powder loading decreases the enthalpy of fusion. Furthermore, the TGA results were independent of the feedstock formulation, whereas the DSC results relied on the feedstock formulation and required comparison to a calibration curve. As a result, the TGA is better suited to determining the consequences of segregation.

Trad et al. [36] also utilized the TGA to evaluate the weight percentage of residual powder following binder burnout at three distinct locations inside the green parts. Powder size, powder shape, and time spent in the molten state can affect powder segregation. It was observed that all feedstocks with powder loading values outside the 60.0 ± 0.25 vol.% range (i.e., nominal powder loading) could be classified as separated mixtures, according to the findings. All powder loading values above or below 60 vol.% represent a powder-rich or binder-rich zone.

To gain a better understanding of how feedstocks are processed, Wei et al. [79] evaluated the coefficient of volumetrically thermal expansion (CTE) of Al_2_O_3_ feedstock using the pressure-volume–temperature (pvT) relationship. At the phase transition point, the CTE value, the slope of the pvT curve, changes. Defects like cracking caused by significant shrinkage in parts can be predicted by determining this value. It is essential to interpret pressure variations in the cavity and at the gate.

## 4. Injection Molding

After the feedstock is prepared, it is ready to be shaped into a useful specimen; the injection molding step accomplishes this. Due to the characteristics of the feedstock used in LPIM, the injection molding machine and the molds used are different from the ones used in HPIM. This section describes the differences in the injection molding step in LPIM and HPIM (Section 4.1). The relationship between the injection molding parameters and defects on the molded parts is discussed in Section 4.2. Finally, the injection molding simulation work for LPIM materials is summarized in Section 4.3. The injection molding step is the fastest in the overall LPIM process, but it is the most important one since the shape given in this step is the shape of the final part and defects in this step are hard to correct in subsequent operations and postprocessing. Therefore, Section 4 aims to provide the necessary information to prevent defects in molded parts and the tools available for molding and setting optimal injection molding parameters.

### 4.1. Injection Machine and Mold

John and Isaiah Hyatt, two brothers, received the first patent for an injection molding machine, a device that used a plunger injection technique to fill cavities, in 1872. Since this version of an injection molding machine operates under low pressure during the injection step, this technology is an example of LPIM [139]. Examples of materials suitable for LPIM are ceramic or metallic compounds with fluidic characteristics and a low viscosity between 1.5 and 4.0 Pa·s. Due to their low viscosity, the transfer into the mold can occur with compressed air and a pressure of 0.8 MPa. Therefore, a complex hydraulic unit, pistons, and spindles are not essential as with the conventional HPIM machine. Other main advantages of the LPIM are low energy consumption, using simple hydraulic mechanisms, small size and lower equipment costs, lower mold wear, lower contamination of the mixture from spindle or piston wear, minimal mixture adhesion to mold, and non-separation of polymer from metallic powder. An LPIM machine is built with an electric heating tank, a mixing and stirring mechanism for preparing the binder system, a vacuuming system, and a unit for casting under pressure by applying compressed air. An LPIM machine is suitable for laboratory tests and production since, on the one hand, it has no moving parts such as screws. On the other hand, large production quantities are possible due to additional tanks for feedstock preparation [88,93,98].

**Figure 4:**
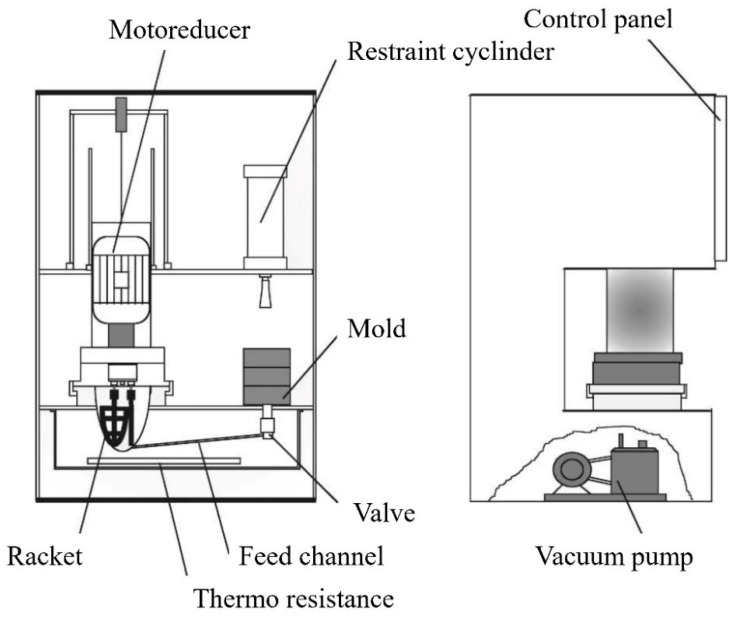
Schematic illustration of LPIM machine. Reproduced with permission from reference [141], copyright © 2001 Elsevier Science B.V.

New concepts for LPIM machines have been established and developed in recent years. A system design having a conical container with heaters was presented by Peltsman and Peltsman [140] 30 years ago, which was suitable enough to maintain the feedstock in the container in a molten state. Similar ideas for injection molding have been put forth by Goncalves [141]. After homogenizing and de-aerating the feedstock, the container is pressurized to allow the feedstock to flow through an interconnection pipe and an injection valve into the mold cavity. Figure 4 depicts this mechanism in illustrative form.

The approach of Nishio and Kawashima [142] involves supplying the mold with feedstock through a pipe while transporting the feedstock without air pressure but with a pump attached to the outlet of a feedstock container. The flow of feedstock through the pump, the container’s input, and the mold’s gate can all be managed by valves. Yamada and Saito [143] proposed a concept for an LPIM machine with a proportional pump connected to the outlet of a raw material hopper and supplying the mold with a constant amount of raw material via a feed pipe and barrel to prevent air from being trapped in the raw material during the injection. Ivzhenko et al. [144] modified an injection machine using a screw pump to transport the feedstock from the hopper to the injection plunger. The mixture is injected into the mold cavity at an injection pressure of up to 20 MPa. This method is suitable for micro/nano-size PIM. Keizo et al. [145] proposed an injection molding machine combining a standard system with a second-stage pressure piston. Goceram AB Corporation [146] used a similar piston system for the medium-pressure powder injection, where the powder-binder mixture flows through a connecting tube from the container to the injection cylinder. The piston of the injection cylinder is then used to convey the starting material into the mold cavity. Lamarre et al. [35] developed an innovative concept for LPIM in which segregation and dead time can be prevented. For this purpose, the interconnecting pipe is eliminated, so strong segregation of the low-viscosity feedstock cannot occur.

Compared to HPIM, low-cost materials such as steel, aluminum, and brass can be used for molds due to their low temperatures and low pressures. According to different studies, silicone molds are also suitable for LPIM because they are reusable and cheap [147,148,149].

### 4.2. Parameters and Defects

The injection molding parameters, i.e., injection pressure and temperature, injection speed, holding time and pressure, and mold cooling, strongly influence the final properties and processing efficiency. The filling is the most critical step in the LPIM process since it is susceptible to flaws, including voids, sink marks, blasting, dead zones, warpage, welding lines, and cracks. Most problems might be prevented if the LPIM procedure were carried out under ideal processing conditions in a well-built mold, using an appropriate feedstock. The feedstock, part size and form, and mold design must all be considered when choosing the parameters [150,151,152].

Due to the higher amount of feedstock to fill the mold, the material compression and density of the green component are improved when the injection pressure is increased. In addition, the green part shrinkage is simultaneously decreased. LPIM feedstocks are typically injected at pressures between 0.7 and 20 MPa (7 and 200 bar) in many studies [32,33,35,37]. The amount of pressure that can be increased depends on the viscosity, but in practice, only an increase between 0.5–0.7 MPa is feasible. The injection speed as a parameter will not significantly affect the part quality. Although at speeds between 25 and 100 cm3/s, the compaction can be enhanced. Based on density measurements, it is evident that lower injection speeds should be used for feedstocks with higher temperatures because their viscosities have been sufficiently reduced. Due to several feedstock constituents, the processing temperature is a critical LPIM parameter. The processing temperature of 90 °C for the feedstocks, including only waxes without any backbone, reduces porosity and shrinkage. In addition, the temperature difference between mold and feedstock in complex mold geometries must also be considered. A heated mold can lead to a reduction of mechanical stresses. If the temperature difference between the feedstock and the mold is minor (slow hardening), PW migrates from the inner to the surface, forming big crystals. Therefore, the outer layers may contain more PW than the layers inside the part. If the temperature difference is large enough, rapid hardening will form tiny paraffin crystals. In this case, PW moves slowly; the injected body has a more uniform paraffin distribution and less volume change, resulting in higher density and mechanical properties [125].

**Figure 5:**
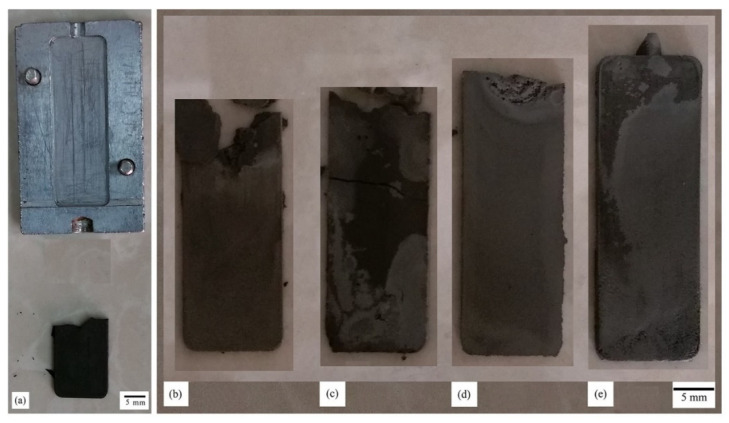
Injection molded parts: (**a**) 60 vol.% powder loading at the feedstock temperature of 165 °C; (**b**) 53 vol.% powder loading at the feedstock temperature of 150 °C; (**c**) 53 vol.% powder loading at the feedstock temperature of 155 °C; (**d**) 53 vol.% powder loading at feedstock temperature of 160 °C; (**e**) 53 vol.% powder loading at feedstock temperature of 165 °C. Reproduced with permission from reference [74], copyright © 2020, Elsevier B.V.

Shahidi Moghadam et al. [74] investigated the fabrication of titanium parts from dehydride titanium powder using LPIM. For this purpose, three powder charges were compared in the study. In order to produce a defect-free green part, the parameters 7–10 bar and 150–165 °C were selected. The mold temperature was chosen as 60 °C, and the injection time of 8 s. The results showed only one defect-free green part with 53% powder by volume when the temperature was 165 °C. In addition, it has been observed, as shown in Figure 5, that short shots occur at higher powder loading, such as 60 vol.%, even if a high temperature of 165 °C is used. Short shots can arise when using low mold temperature and injection temperature, although too low injection pressures also could result in incomplete mold filling. Due to low flowability, solidification occurs before the entire mold is filled in a short shot.

**Figure 6:**
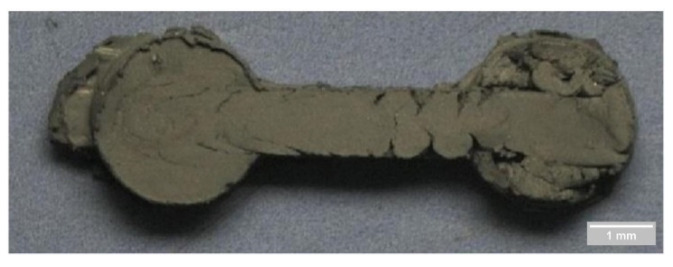
Jetting problem during the injection molding step. Reproduced with permission from reference [33], copyright © 2014 Elsevier B.V.

Fayyaz et al. [33] studied the LPIM of cemented tungsten carbide components. Due to high pressures exceeding 12 bar combined with high fill rates, jetting happened during the injection testing. The defect has been shown in Figure 6. In LPIM, jetting and flow lines, according to Piotter et al. [22], can be avoided when the pressure is lower than 10 bar. However, insufficient feedstock feeding into the mold occurs if the pressure is too low during the injection. Therefore, the individual parameters must be set precisely to produce defect-free parts. The selection of proper injection molding parameters should be considered to reduce defects and failed parts because the imperfections in the green part may introduce some problems in the debound or sintered part [153,154,155].

The molding parameters significantly influence the quality of the injected components. However, irregularities during feedstock preparation cannot be corrected during injection molding [156,157]. In summary, mold design and feedstock quality significantly impact the injection parameters. This leads to the conclusion that not one collection of circumstances is thought to be sufficient for the molding process. Injection times, for instance, varied from 5 to 60 s with a mold filling speed of 1.5 cm^3^/s [42].

**Figure 7:**
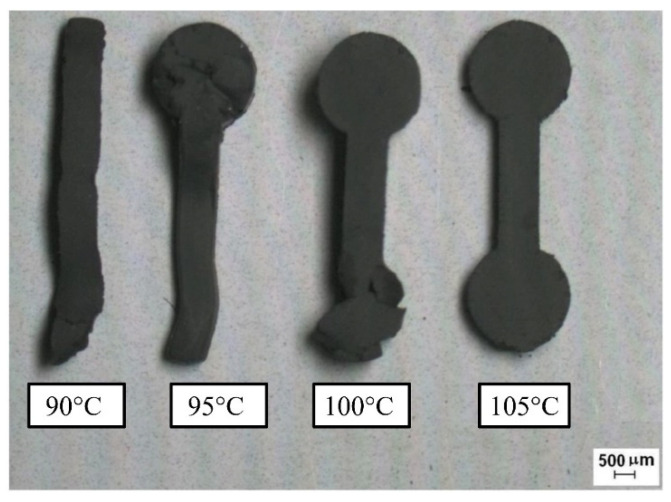
Green parts at different mold temperatures. Reproduced with permission from reference [33], copyright © 2014 Elsevier B.V.

The mold temperature significantly impacts the injection step and subsequently influences the following stages. According to Fayyaz et al. [33], the optimal injection parameters were 110 °C injection temperature, 10 bar pressure, 7 s injection time, and the mold temperature of 105 °C. The results showed that mold temperature significantly influences the injection molding quality (Figure 7).

**Figure 8:**
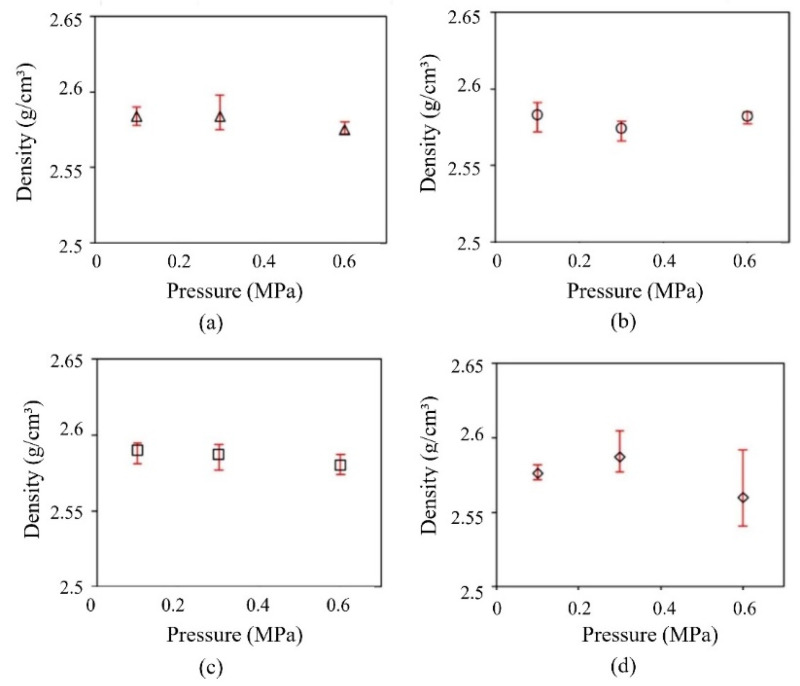
Effects of the pressure and temperature on the density of the green parts: (**a**) 70 °C; (**b**) 80 °C; (**c**) 90 °C; (**d**) 100 °C. Reproduced with permission from reference [86], copyright © 2016 Elsevier Ltd.

According to the research by other authors [158,159], the defects that surfaced during debinding or sintering were not always caused by those steps but could instead have their roots in injection molding. Molding flaws can be fixed by changing the molding parameters. Despite having no apparent imperfections, molded objects may still experience dimensional variation or distortion during sintering because of their density gradient. Sardarian et al. [86] optimized the LPIM process parameters to fabricate alumina ceramics. An alumina feedstock with 60 vol.% powder was injected into the mold at temperatures and pressures of 70–100 °C and 0.1–0.6 MPa, respectively. Except for 70 °C and pressures less than 0.3 MPa, these temperatures and pressures were sufficient to fill the cavity. According to the findings, raising the injection temperature and pressure has a beneficial impact on how well the mold cavity is filled. Figure 8 shows the effect of the process parameters on the green density in this study. The green density of the components is not significantly impacted by either the injection temperature or pressure. The variability of the obtained density increases since the error bars are larger at higher temperatures.

Utilizing injection molding at a temperature of 80 °C and a pressure of 0.6 MPa produced the best results. Variation in the green density of the components may have occurred because of void formation during the filling step when the parts were molded at high injection temperatures and pressures. The occurrence of jetting may be responsible for the void’s creation. The shrinkage linked to the brittle nature of the green parts is another crucial element of the LPIM process. As previously indicated, waxes are frequently added to LPIM feedstock to decrease the viscosity. Too much wax content in the feedstock can cause shrinkage when the injection mold cools and the part solidifies. Because of the range of applied pressure, approaches like the overshot are not feasible (usually under 0.7 MPa). The feeding gate typically hardens quickly, making pressure control challenging.

Costa et al. [88] investigated welding line formation in holes obtained by LPIM of ceramic parts. Injection molding and welding lines typically form due to the material flow being divided by an obstruction and its posterior junction [160,161,162]. Welding lines reduction is possible by using a low percentage of the waxes in the feedstock. In LPIM, which requires subsequent debinding and sintering, welding lines might cause stress concentration and crack formation.

### 4.3. Simulation

Mold filling is one of the most crucial processes in the PIM process that must be controlled to produce a complete mold filling and to avoid defects like voids, welding lines [163], jetting, warpage [164], fractures, and sinks [165].

Simulations and models used for plastic injection molding have also been applied to PIM. Nevertheless, the high powder content frequently results in changes that the models used in plastics neglect. Several cases exemplify the issues, including powder–binder separation at welding lines, significant inertial effects in the molding of tungsten alloys, and fast heat loss as in the molding of copper and aluminum nitride. Additionally, powder–binder combinations are extremely shear-sensitive. As a result, the computer simulations used to assist molding in the plastics industry need to be improved to work for LPIM. The improvements should include those feedstock characteristics in new PIM models for filling, packing, and cooling [166,167,168,169,170,171]. To optimize the process parameters and eliminate molding flaws, numerous physical characteristics, such as the material filling times, flow behavior, and injection pressure, have been effectively predicted using numerical simulations [172]. For metallic feedstocks, the injection stage has been modeled mostly for high-viscosity powder binder mixtures designed for the HPIM [1,168,173,174,175,176].

Simulations of mold filling, including jetting, filling time, and pressure distribution, were primarily done for ceramic-based feedstocks [85,177] in the LPIM process. However, few numerical models describing the injection stage for metallic-based LPIM feedstocks have been identified in the literature [111,116].

For example, Ghanmi and Demers [116] determined the moldability potential of a complex-shaped specimen made from titanium-based feedstock through numerical simulations. This study’s objective was to evaluate the effect of low-viscosity binder components on the moldability of LPIM titanium-based feedstocks and to anticipate the occurrence of defects using numerical simulations. They applied Moldflow to detect the high shear rate zones encountered by the feedstock, where laboratory testing confirmed the predicted segregation of 2 vol.%. However, computational models did not accurately anticipate the halo-shaped segregation pattern evident in the cross-section of the injected parts. In another study for metals, Azzouni et al. [111] examined the feasibility of simulating the mold-filling behavior of a 17-4PH stainless steel feedstock with the LPIM using the commercial program Autodesk Moldflow Synergy 2019. Since actual and simulated injections were conducted at a constant volumetric flow, the injected length and melt front velocity were not affected by the feedstock temperature but rather by the geometry of the mold cavity. In this regard, the injection length and melt front velocity predicted by the numerical model were in excellent agreement with experimental results, with a maximum relative divergence of 0.5% and 3.7%, respectively. In addition, they determined that the numerical model almost accurately represented the drop in injection pressure with an increase in melt temperature or an increase in mold cavity cross-section for filling stages below 75%.

Using Moldflow Synergy, Sardarian et al. [84,85] predicted the filling time and pressure for an alumina-based feedstock used in LPIM. The same study team also demonstrated Moldflow Synergy’s remarkable ability to comprehend better the impact of pressure, temperature, and flow rate on the jetting phenomena and improve injection settings [84]. Ben Trad et al. [37] proved Moldflow’s capacity to replicate the melt front velocity, injected length, filling time, and segregation phenomena in LPIM. The simulated injection pressures achieved by prior research teams have never been confirmed with actual measurements. Their study aims to validate the Moldflow modeling tool’s capacity to estimate the in-cavity pressure during the injection phase of LPIM metallic feedstock.

As previously mentioned, the incomplete filling is one imperfection typically resulting from a lack of fluidity in the feedstock or a too-narrow mold cross-section. Other causes of this problem include resin oxidation, improper injection-molding temperature, inadequate shot volume, and a low feedstock flow rate. Before solidification, the feedstock must fill the mold during the filling stage. The processing temperature and polymer flow rate may be excessively high to minimize unfilled regions, leading to more burr defects [178]. In this context, Yavari and Khorsand [112] conducted numerical and experimental research on separation, injection step, and imbalance filling in LPIM of ceramic components. SiC feedstock was injected at varying temperatures and flow rates in this study. It was determined that the segregation and imbalance filling phenomena are more pronounced in thin-walled samples, but imbalance filling was not found in flexural specimens. Nevertheless, the thin-walled sample is considerably more sensitive to changes in flow rate and temperature. Thus, increasing the temperature, since the viscosity decreases, the binder flows ahead, and the solid loading is reduced. The separation phenomena enhanced as temperature and flow rate increased. Imbalanced filling, filling pattern, experiment findings, and segregation simulation were consistent.

## 5. Debinding

The goal of the LPIM process is to obtain parts made of metal or ceramic. Thus, the organic binder must be removed entirely from the shaped specimen in the debinding step. Debinding is the most time-consuming step in PIM technology since it is a highly complicated procedure involving the simultaneous operation of several diverse processes. In a successful debinding procedure, the binder components are extracted from the injection-molded specimen or green part without disturbing the powder particles and the overall geometry of the specimen. Before proceeding to the higher temperatures necessary for sintering, all binder components must be eliminated to avoid contamination of the metal or ceramic. Binder removal is possible since the sintering temperatures are significantly higher than the binder volatilization temperatures [179,180,181,182].

Consequently, any leftover binder quickly escaping from the component may cause the part to fracture, lose its shape integrity, or produce soot, which may alter the material’s composition and, thus, its intended performance. Debinding is more critical for LPIM feedstock without backbone or with a minor amount of backbone since the part is more fragile. Frequent debinding techniques include solvent debinding and thermal debinding [183,184,185,186], which are discussed in Section 5.1. In lacking a backbone binder for some LPIM feedstocks, shape retention is one of the process’ most significant challenges; thus, an alternative method for debinding in LPIM is thermal wick debinding, in which all binder components are eliminated in a single operation in a bed of loosely packed powder. More details of this type of debinding are described in Section 5.2 below.

### 5.1. Solvent and Thermal Debinding

Solvent debinding is performed by immersion of the molded part in a gaseous or liquid solvent such as hexane, heptane, ethanol, or acetone at a specific temperature [182]. When the main binder, also known as the soluble binder, is removed, a porous network is generated. This interconnected porous network allows the volatilizing polymer to exit without breaking, deteriorating or producing abrupt stresses that damage or distort the molded object [187]. Since the utilized solvent must enter the specimen, the geometry of the green component, particularly its surface-to-volume ratio, is a significant element in determining the speed of solvent debinding. Temperature and porosity also impact the solvent debinding procedure. As the temperature rises, the interaction between the soluble binders and solvent will intensify, meaning that the temperature effectively modifies the solubility and diffusion coefficients [188,189,190]. Note that the time necessary for debinding depends on the debinding process, the binder system, the dimension of the part, the particle size, and the distribution of the powders used to produce the specimen.

No diffusion bonding between powder particles occurs at low temperatures when the major binders are eliminated. After the primary binders have been eliminated, the secondary binders, also known as backbone binders, with the help of interparticle friction, could keep the powder particles together and retain the injection-molded shape. The secondary components of the binder system are removed thermally. This is done by heating the parts to their degradation temperatures and keeping them until all binders turn into gases. If more than one secondary binder is present, more temperature holds may be necessary [101].

There are several causes for incomplete binder removal, but they all come mainly from incorrect debinding temperature or inadequate debinding time at a decomposition temperature. In debinding, the gas flow rate is also a significant issue. Insufficient gas flow inhibits the removal of gaseous binders and causes them to remain within the component. This has the same effect as removing the binder at an incorrect temperature [191,192,193,194].

Gas flow is a processing factor that depends on the binder system and composition, as well as the particle size and dispersion of the powder. The residual binder is expelled at temperatures higher than it should have been eliminated, and the heating rate is also accelerated. In this instance, fast gas formation may result in flaws such as cracks, blisters, and contamination that alter the chemical composition. Because there is only a tiny amount of backbone in some LPIM feedstocks, it is anticipated that the debinding process would provide more challenges than the HPIM process. However, it is still possible to use solvent and thermal debinding in two steps [112].

Shahidi Moghadam et al. [74] performed LPIM on titanium components using two phases of debinding for a binder containing PW, LDPE, and SA. During the solvent debinding stage, the solvent diffuses into the interlayer more efficiently at higher temperatures than at lower temperatures, resulting in a quicker dissolution into solvent and an increase in the rate of solvent debinding. At 75 °C, the rate of binder removal was relatively high. However, the solvent debinding procedure ended after 60 min due to the observation of component fractures after debinding at this temperature. These findings are comparable with those of Zaky [195], who discovered that a high temperature resulted in cracks in the molded component upon extraction. It is probable that the extremely rapid extraction rate caused the cracks at 75 °C and that the LDPE backbone was softened. After solvent debinding at 45 °C and 60 °C, the samples exhibited no defects. However, the binder was removed gradually at low temperatures (45 °C). Therefore, 60 °C was chosen as the optimal solvent debinding temperature.

**Figure 9:**
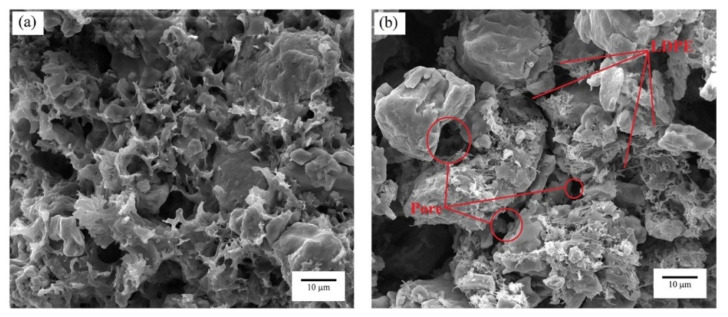
SEM image of samples: (**a**) injected part; (**b**) solvent debound part. Reproduced with permission from reference [74] copyright © 2020 Elsevier B.V.

Figure 9a,b are SEM images of the molded and solvent debound components, respectively. As shown in Figure 9a, the powder and binder were thoroughly kneaded, indicating that the powder-binder combination was homogenous. Figure 9b demonstrates that the soluble part of the binder system was removed, leaving the structure with pores and open channels. This is advantageous for extracting LDPE from solvent-debound portions during thermal debinding since the LDPE decomposes and diffuses from the interconnected channels. Figure 9b demonstrates that the LDPE stayed in components and linked powder particles to preserve adequate strength for the thermal debinding and sintering processes.

**Figure 10:**
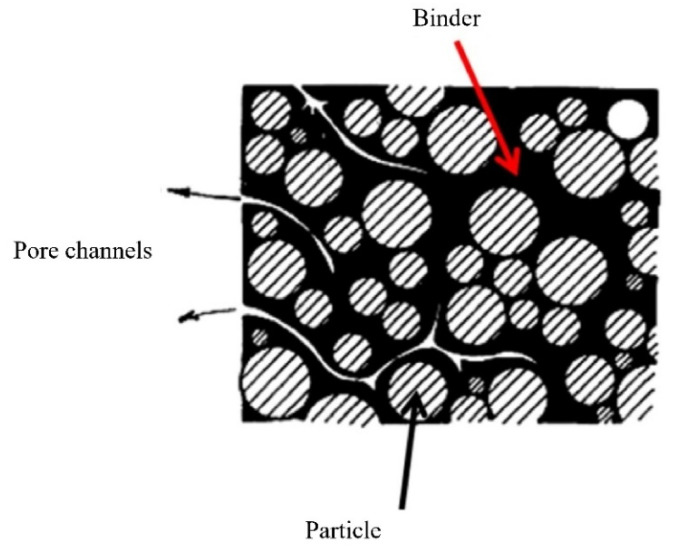
The schematic of binder distribution during the solvent debinding process. Reproduced with permission from reference [42], copyright © 2016 Elsevier B.V.

Large cross-sectioned components manufactured from submicron-sized ceramic powder provide significant challenges during binder removal [74]. Measuring the effectiveness of binder removal requires removing the binder without compromising the cohesiveness of powder particles. As illustrated in Figure 10. Hsieh and Hwang [196] asserted that solvent debinding begins at the sample’s surface and progresses inward to the core, leaving pores behind. Raza et al. [197] found that an improper thermal debinding cycle increases residual carbon, eventually weakening sintered parts’ corrosion resistance and mechanical qualities.

**Figure 11:**
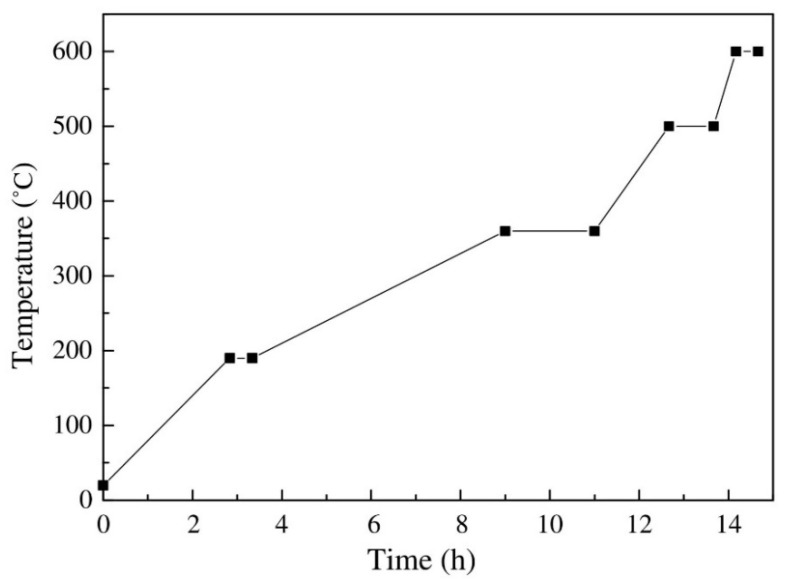
Debinding cycle of 14.5 h to remove EVA and LDPE. Reproduced with permission from reference [97], copyright © 2004 Elsevier Ltd.

Optimization of the debinding schedule has attracted several theoretical and practical efforts for decades [198,199]. Zhang et al. [97] utilized two thermal debinding procedures for the feedstock consisting of AlN–TiB2 powder along with PW, LDPE, EVA, and SA as the binder system. The 31-h procedure did not detect any macro-defects in the final goods, as seen in the graph. For the binder constituents with high melting points to be successfully removed at higher temperatures without deformation or cracking, it is necessary to manage a modest degradation rate in the first stage. The initial degradation temperature of the binder decreases with decreasing heating rate, and the TGA curve goes in the direction of low temperature, as previously described. Figure 11 depicts the debinding program with a higher heating rate and a total binder burnout time of 14.5 h.

**Figure 12:**
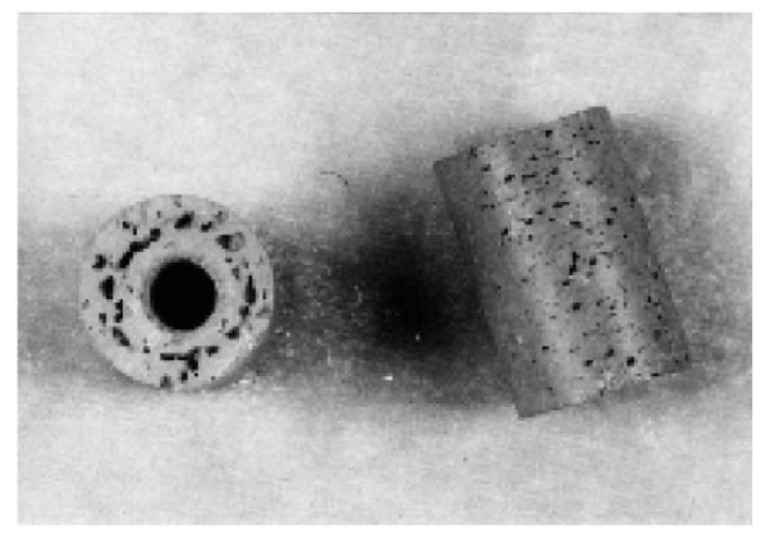
Sintered parts with many pores due to quick thermal debinding. Reproduced with permission from reference [97], copyright © 2004 Elsevier Ltd.

Consequently, 190 °C was the beginning of the decomposition and the holding temperature. The heating rate of the subsequent stage before 500 °C is quicker than the preceding step. Finally, the green components are pre-sintered at 600 °C. After sintering, the specimens exhibit many pores, particularly in the middle of the pieces. Figure 12 depicts the sample after sintering. The reason for the emergence of pores in the parts is that the heating rate is so rapid that a large amount of volatile degradation production is generated simultaneously in the whole component. In addition, the degradation products in the center of the part cannot be removed in time, resulting in the formation of bubbles in the specimen. Thus, it is evident that the debinding period must be extended. Several temperature preservation stages were added to the debinding program to lengthen the duration during which PW is gradually removed to decrease debinding defects.

**Figure 13:**
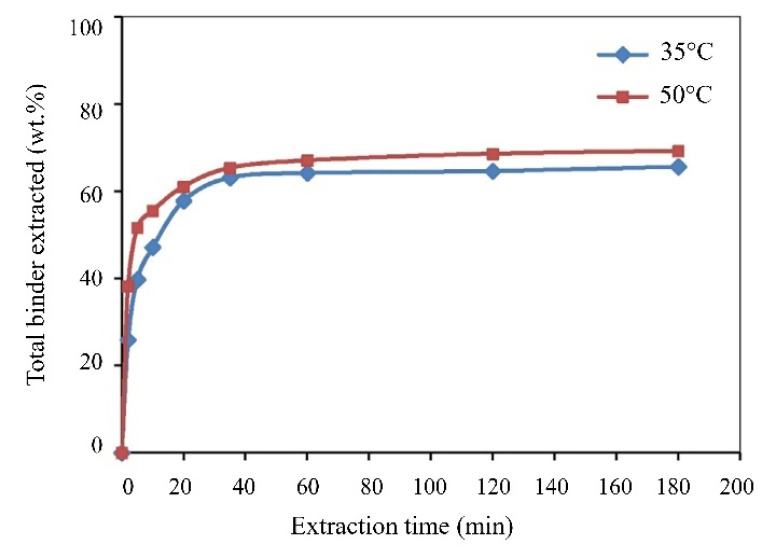
Effects of debinding temperature and time on the amount of the binder system removed during solvent debinding. Reproduced with permission from reference [33], copyright © 2014 Elsevier B.V.

In another investigation, Fayyaz et al. [33] did a two-stage debinding, including solvent and thermal debinding. In the stage of solvent debinding, PW and SA were eliminated. The molded pieces were submerged in n-heptane for 3 h at two different extraction temperatures (35 °C and 50 °C). The extraction rate of the binder by solvent debinding was determined by measuring the mass loss of at least five specimens subjected to identical circumstances. Thermal debinding was carried out under argon at a heating rate of 5 K/min to 350 °C, followed by a 15-min dwell, then at a heating rate of 3 K/min to 490 °C, with a 2.5-h stay. Figure 13 depicts the amount of binder removed with time throughout the solvent debinding process at various temperatures. After 3 h, 65 and 69 wt.% of the binder components were removed at 35 °C and 50 °C, respectively. Due to the increased diffusion rate, higher temperatures result in a quicker binder extraction. When the temperature is increased to 50 °C, the rate of binder extraction rises, but some samples develop fractures. This cracking can be due to the rapid pace of binder extraction at 50 °C. When the binder was extracted slowly at low temperatures (35 °C), there were no fractures in the molded component, and virtually all the soluble binder was effectively removed.

### 5.2. Thermal Wick Debinding

In the case of some LPIM binder systems, debinding is particularly challenging since the binder consists simply only of wax or just a small amount of a polymer backbone that would hold the particles together during thermal debinding [200]. When the wax binder in LPIM-shaped components melts during thermal debinding, only weak attractive forces hold the powder particles together; hence, even slight stresses can result in irreversible deformation [92]. Solvent debinding is challenging to apply to LPIM because the binder is composed primarily of PW. Wick debinding is a highly successful method for preventing the creation of LPIM faults [201,202].

Binder removal typically occurs in two sequential steps. The first step extracts the lower melting binder components at temperatures slightly below their boiling point. This stage in LPIM deals with the reversible melting of waxes and comparable organic substances. Therefore, the binder removal must be carried out securely, avoiding warping the molded component and destruction due to incorrectly removing the employed binder system. In order to prevent these unfavorable variables, debinding is often carried out in an absorbent, i.e.,; the molded bodies are submerged in a ceramic powder that supports the safe removal of the binder by absorbing molten organics via capillary forces during heating [125]. The absorbent supports the components, while the melting PW-based binder or backbone inhibits component deformation. This method has been described and optimized for industrial use [203,204]. Debinding without powder absorbent is only achievable for tiny components with a maximum height of 10 mm and a maximum wall thickness of 5 mm when put on porous setters that absorb molten organics. To perform debinding without absorbent, the thermoplastic binder level in the feedstock should ideally be 10 vol.%. This can be lengthy; however, it is significantly accelerated by placing the compacts on a bed of loosely-packed powder. As the wicking embedment, a very porous oxide ceramic powder or granulate, such as alumina, is employed in practice. The finer particles are a wicking agent, pulling the binders out of the green component by capillary action. After this step, the porosity should ideally be interconnected, and the compact should be reasonably permeable. The heat breakdown of the residual binder is the second phase [90]. Here, the residual components of the binder are evaporated and depolymerized through heat. The gases created during depolymerization rapidly pass through the porous, open structure. The minimum compact thickness, the packing percentage, the binder composition, and the pore size are the primary variables that must be addressed while constructing an optimal debinding cycle. Because thermal degradation and evaporation create gases, current thermal techniques need a prolonged thermal debinding step with heating rates between 5 and 10 K/h to prevent the formation of bubbles and flaws in the compact. However, with the wicking approach, most binders are removed in the liquid condition, resulting in fewer flaws and a shorter time to remove the remaining binders [90].

Due to the migration and softening of the thermoplastic binder components, the powder particles also begin migrating and rearranging their location, which is another crucial aspect of the need for a slow debinding process. A gradual debinding process improves the rearranging and compaction of particles during migration. Liu et al. [205], who studied the debinding of HPIM (PW and EVA were used as the primary and secondary binder system components), emphasized the significance of particle mobility on densification; this characteristic is also relevant to the LPIM. Some researchers have used wick debinding in the LPIM. For instance, Gorjan et al. [100] performed partial wick debinding of LPIM components, which comprised experimental work and a theoretical model. Several researchers have conducted theoretical research into wick debinding [203,204,206,207]. Some of it was based on HPIM tests. However, they are also relatively broad and applicable research to the LPIM. LPIM-typical theoretical work based on tests with a single-component binder has also been undertaken [40].

**Figure 14:**
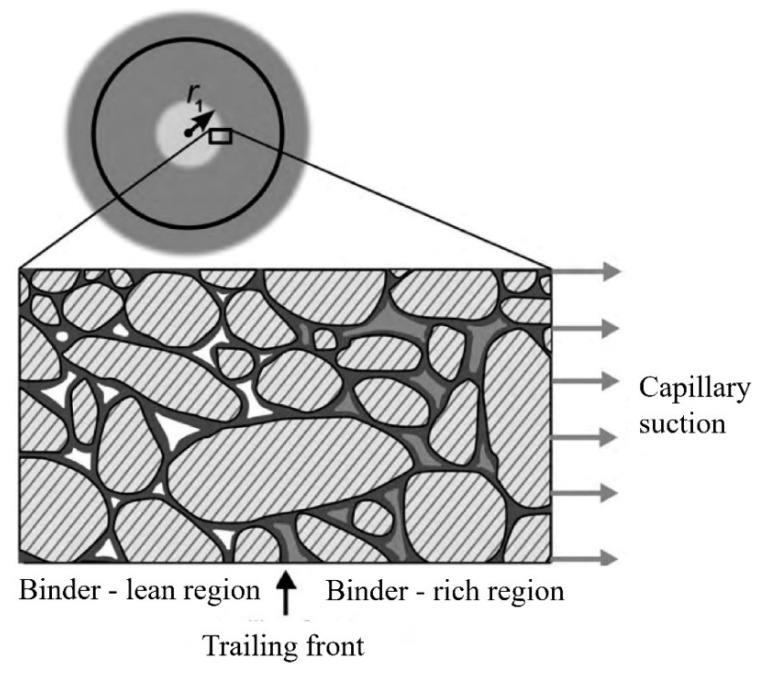
Schematic of the binder system distribution during thermal wick debinding. Reproduced with permission from reference [100], copyright © 2010 Elsevier Ltd.

There are two states for the binder inside the part: as a mobile part of the binder system in the larger spaces between powder particles, which may flow owing to the pressure gradient, and as an immobile part of the binder on the surfaces of the particles and within the smaller voids, which cannot be moved due to capillary suction because it is too strongly attached to the powder (Figure 14). When a body containing molten binder comes into touch with wicking powder, capillary suction draws the mobile portion of the binder toward the wicking agent. The immobile amount of the binder system (dark gray surrounding the particles), which is trapped inside the tiny pores and particle surfaces, remains in its position. This distinction is not based on the chemical composition of the binder but on the location alone. According to this model, only 25% of the mobile portion of the binder may move due to capillary suction. The model also suggests that the flow resistance of the molten binder is significantly higher in the wick embedment than within the molded component. When the binder melts, its density changes, which explains the considerable decrease in binder content at the beginning of the operation. Because the powder structure is rigid and non-deformable, the expanding binder is expelled from the green component. In addition, an oxidation process predominates as the mechanism of binder removal if the temperature during debinding surpasses 180 °C [100].

In another study, Gorjan et al. [40] executed single-step wick debinding and sintering of alumina for LPIM. The process goes from molded to sintered components in about 25 h. This was accomplished by using a high-purity carbon black as a wicking embedment, which burns off once that function as a wicking agent has been fulfilled. The bending strengths of the sintered samples suggest that the carbon black’s burnout does not affect the final characteristics. The LPIM process might greatly benefit from the proposed method.

The embedment not only plays an active role in the debinding via capillary suction of the molten binder [201,208,209], but it also provides physical support for the softer sections while melting the binder system. Capillary extraction is required to produce high-quality LPIM components, unlike HPIM, where the binder may be removed in a gaseous phase without embedment [210,211,212]. Due to its thermal stability, it may be cleaned repeatedly by burning the leftover binder at a high temperature [213]. With capillary extraction, a large portion of the binder may be removed, resulting in porous sections from which any remaining binder can be entirely removed in the gaseous phase during heating to the sintering temperature.

Tafti et al. [101] studied the influence of thermal debinding on the LPIM of irregular iron-based feedstock using a binder combination of PW, SA, and EVA at a 55 vol.% powder loading. Three pre-sintering temperatures were used for the wick debinding process (600, 700, and 850 °C). According to the findings, increasing the pre-sintering temperature from 600 to 850 °C reduces the incidence of fine loose powder particles dispersed inside the debound network. It encourages bonding at the surface (dense layer) and subsurface (particle bonding). The drop in sintered density from 6.2 to 5.1 g/cm^3^ observed with a rise in pre-sintering temperature suggests that this debinding parameter is significant and should be kept as low as feasible during thermal wick debinding. In this regard, thermal wick debinding at a low pre-sintering temperature resulted in no evident bonding on this irregular iron powder. The most critical parameters in thermal wick debinding are debinding temperature, the wicking medium pore size, pre-sintering temperature, heating rate, and protective atmosphere. All must be set to ensure proper binder extraction while avoiding undesirable stresses, distortions, defects, or chemical reactions [214].

German [215] pioneered this subject, describing the mechanics driving thermal wick debinding and the binder’s two different structures via the powder network. On the one hand, capillary forces take a large quantity of the molten state binder from the injected components as a connected liquid (also known as the funicular state) within the highly permeable wicking bed. While on the other hand, the binder regions get segregated, and the leftover binder stays trapped within the component (also called the pendular state). The binder is degraded during a heating ramp-up to pre-sintering temperature regardless of location. According to this concept, the binder content should evolve at different stages, with the center of the part becoming poorer and the wicking bed becoming more affluent in the binder.

In contrast with this concept, which divides the binder system during debinding as immobile or mobile sections, further experimental data provided by several research groups revealed a few discrepancies with this idea. Experimentally, Somasundram et al. [204] investigated the processes of thermal wick debinding for a zirconia-based LPIM commercial feedstock. During the debinding, a uniform distribution of the binder was observed across the components. They determined that the binder was removed simultaneously from larger and smaller holes in the feedstock, irrespective of the position. In addition, Kim et al. [216] investigated the influence of additives on the capillary extraction forces during thermal wick debinding treatment using a commercial silicon nitride-based LPIM feedstock. In terms of capillary extraction, weight loss, and debinding rate, they discovered that binder systems comprising EVA performed differently from those employing PE wax. The study also concluded that the transition from the funicular to pendular states and the capillary structure are greatly influenced by binder homogeneity, independent of binder placement within the component.

## 6. Sintering

The last step of the LPIM process is sintering. The debound component is transformed into a dense body during the sintering step, typically performed in a sintering furnace, where it occurs in steps. Sintering in LPIM is similar to that of HPIM, and it is a thermal treatment procedure that coalesces particles by expending surface energy, improving the characteristics of molded parts [42,187,217,218,219,220]. Typically, sintering occurs at 70–90% of the melting point [221]. Sintering is conducted in a vacuum, nitrogen/hydrogen, or hydrogen environment to prevent powder oxidation and depends on the chemical composition of the powder. During sintering, considerable atomic diffusion and chemical changes to the outer surface occur when the component’s temperature reaches approximately 1/2 to 2/3 of its melting temperature. Depending on the powder composition and shape, solidification can be between 95–99% of the theoretical density [222,223]. Recrystallization and grain growth follow atomic diffusion in the solid state when the sintering procedure is carried out.

**Figure 15:**
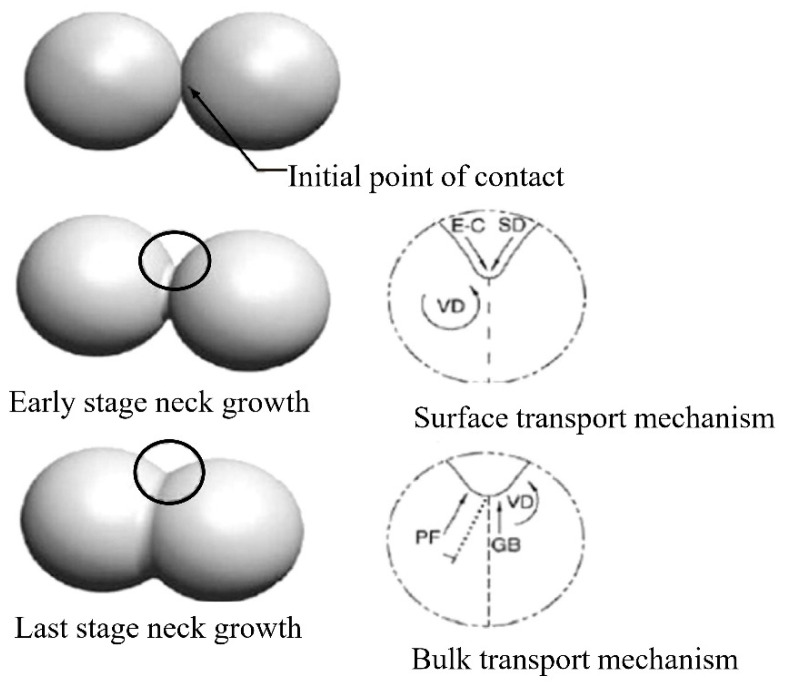
Two-sphere sintering model. Reproduced with permission from reference [42], copyright © 2016 Elsevier B.V.

As the temperature rises, thermolysis occurs, which aids in burning away any leftover polymeric material from the binder. As the sintering temperature approaches the material’s melting point, atomic diffusion and specific chemical reactions will eventually begin. Sintering theories suggest the process happens between two particles close (in contact) under isothermal conditions [224,225,226]. Figure 15 depicts a bi-spherical sintering model with contacting spheres. Due to mass transfer, a neck forms and then expands between both particles. As evaporation and condensation move atoms to the surface, volume and surface diffusion occur. Consequently, two spherical particles stay stationary during sintering, as seen in the last step of the necking phenomenon; the atoms aid neck development and increase the bond length between the spheres by volume diffusion, grain boundary diffusion, and plastic fusion (Figure 15).

Consequently, the consolidation of powders increases their strength. Depending on the powder utilized and the powder loading, the linear shrinkage of sintered components ranges from 15 to 22% [227,228]. Material and process factors determine the sinterability and microstructure of a sintered powder compact. In addition to the powder’s size, particle size distribution, and agglomeration, the powder and the chemical composition of the powder compact is also a material variable. These eventually affect the grain’s densification and growth.

On the other hand, the pressure, temperature, atmosphere, time, and heating and cooling rates are typically referred to as process variables and are frequently denoted as thermodynamic variables [229].

As previously mentioned, the sintering of LPIM components is very similar to the sintering of HPIM components, therefore the literature to determine the influence of sintering temperature and atmosphere on LPIM sintered components is sparse. Therefore, the reader is referred to books to learn about the sintering parameters needed for different metals and ceramics [16]. The main factor that differentiates LPIM from HPIM is the amount of pressure used during the injection molding step and there have been investigations into the effect of injection pressure on green density [79], which is relevant to this review paper. The results indicate that higher pressures may enhance the density of the green parts, which is advantageous for the sintering step since particles are closer to each other and sintering relies on particles being in contact (Figure 15).

Due to extremely low pressures during injection molding, sintering in LPIM might be difficult and the use of sintering additives might be necessary. For example, Yavari and Khorsand [113] investigated the effect of additive content and sintering temperature on the mechanical properties of clay-bonded and glass-bonded ceramic parts produced by LPIM. In this study, porous silicon carbide ceramic was successfully fabricated from SiC, kaolin, and borosilicate glass at different sintering temperatures of 1200, 1300, and 1400 °C in an air atmosphere by the LPIM method. During sintering, the glass frit converted into a viscous glass phase, which operated as a bonding agent between SiC particles and a protective barrier against the severe oxidation of SiC particles. At 1300 °C, kaolin transformed into cristobalite, and the liquid phase sintering increased flexural strength and decreased porosity. With the increasing initial particle size of SiC, the porosity of porous SiC components decreased.

Numerous studies using sintered components have been conducted successfully using the LPIM method but have not explored the effect of varying sintering settings on this method. Therefore, investigating the relationship between employing low pressures and sintered characteristics might be a fruitful study area for the future.

## 7. Summary and Conclusions

LPIM is a developing method that may offer the potential to manufacture small and complex-shaped parts at a lower cost than HPIM. The first step of both PIM processes is feedstock development. The primary distinction between LPIM and HPIM is the composition of the binder, which makes this LPIM more challenging, owing to its unique injection molding and debinding issues. The feedstock formulation affects injection molding and debinding. For example, an appropriate binder system for a given powder can help to mitigate various flaws resulting from using low pressure during injection molding. The binder composition determines the type of debinding that can be used. Most LPIM binder systems lack a polymer backbone that aids shape retention, except for a few metallic feedstocks. Without a backbone, in the feedstock, the most common debinding method is thermal debinding. The thermal debinding method might face several obstacles, some of which can be overcome by wicking thermal debinding.

In this review paper, the differences between HPIM and LPIM in the injection molding stage have also been identified. It can be mentioned that feedstocks’ injection molding of feedstocks for LPIM is more challenging due to low injection pressures and using feedstocks with very low viscosity. This causes many problems, increases the possibility of different defects during the injection, or makes the injected parts susceptible to imperfections in subsequent process steps. However, the lower cost of the equipment, which makes the LPIM a cost-effective process, whether in low or high production volumes, is one of the most significant advantages. A further benefit of LPIM is the ability to do injection molding at lower temperatures, which is favorable for some specific powders like magnetic alloys vulnerable to oxidation at temperatures above 200 °C. This ensures that the properties of green and sintered components can be improved. Similarly to HPIM, the use of numerical simulation of the LPIM process can help determine the processing parameters to obtain the best molding quality and it is highly recommended to design appropriate molds. Compared to HPIM, LPIM allows for one-step debinding, eliminating the need to handle the parts during several debinding phases. This can lessen the chances of forming defects. Less contamination during thermal debinding and complete debinding at lower temperatures are two additional benefits of the LPIM with a single thermal debinding stage. However, a combination of solvent and thermal debinding has been successfully used also in LPIM, which can shorten the debinding time. However, eliminating most of the wax-based binder system by solvent extraction, increases the risk of component defects due to the extra handling of very fragile parts. Solvent debinding also requires the use of a backbone that is not soluble and the use of additional equipment to carry out solvent extraction in a safe and reliable manner.

The sintering step in LPIM is very similar to that of HPIM and it is highly dependent on the metal or ceramic powder present in the feedstock. The correct heating rates and holding times are crucial to ensure the densification of the shaped specimens and obtain the expected mechanical performance. Another critical parameter is the selection of the correct sintering atmosphere to be able to obtain the required microstructure and remove contaminants due to the use of the organic binder and exposure to air. The low pressure used in LPIM can affect the sinterability of shaped components and the use of sintering additives can be sometimes required.

LPIM is a promising technology that already is being used in commercial products. However, there are still further improvements that can make it more reliable, some suggestions are presented in the last section of this review.

## 8. Future Perspectives

Even though several studies and industrial applications have been conducted on the LPIM subject, it requires more optimization and analysis to speed up feedstock development, decrease defects, enhance the final quality and increase the applications of LPIM. In the area of feedstock development, the use of computational tools such as molecular dynamics simulations and artificial intelligence could be used to limit the number of experiments needed to obtain a suitable feedstock for new powder materials and, therefore, speed up the development time. Further computer simulations could decrease defects and increase quality; however, the existing models have yet to accurately predict the flow behavior and powder separation occurring during the injection molding process; therefore, further modifications are needed. Finally, to increase the applications of LPIM, it is necessary to use new powders and identify applications that can benefit from the complex geometries that LPIM can produce.

Using Al, Mg, and magnetic alloys might be one of LPIM’s most remarkable areas of expansion because of the wide variety of applications for these sensitive alloys. One of the problems in developing binder systems for these sensitive alloys is carbon and oxygen contamination; such contamination is detrimental to the final properties of sintered components. Most of the carbon residue comes from the burning backbone in thermal debinding that can result from the overlap of the degradation of binder components used in HPIM and the sintering temperatures of these sensitive alloys. Therefore, binder systems used for LPIM would be a new opportunity to reduce the problems of processing magnetic, Al, and Mg alloys. Furthermore, for specific alloys, such as Mg and Al, with low sintering temperatures, a complete debinding at lower temperatures compared to HPIM is extremely important. Because it is challenging to develop appropriate binder systems that could be removed before the sintering for these alloys in the HPIM, these benefits could present new opportunities.

Another future opportunity is to adapt materials used in LPIM for additive manufacturing techniques that rely on secondary operations for the fabrication of metal and ceramic parts. The most straightforward transition could be to use LPIM feedstocks material extrusion techniques, such as direct ink writing and robocasting since the feedstocks require similar behaviors to those used in LPIM.

## Data Availability

Data sharing is not applicable to this article as no new data were created in this study.

## Abbreviations

BW	Bees Wax
CAB	Cellulose Acetate Butyrate
CIM	Ceramic injection molding
CTE	Coefficient of thermal expansion
CW	Carnauba wax
DSC	Differential scanning calorimetry
EVA	Ethylene-Vinyl Acetate
HDPE	High-Density Polyethylene
HPIM	High-pressure powder injection molding
LDPE	Low-Density Polyethylene
LOP	Licowax OP
LPIM	Low-pressure powder injection molding
MFO	Menhaden Fish Oil
MIM	Metal injection molding
MW	Microcrystalline Wax
OA	Oleic Acid
ODA	Octadecylamine
P2E2O	Poly(2-ethyl-2-oxazoline)
PD	Pycnometer density
PE	Polyethylene
PEG	Polyethylene Glycol
PIM	Powder injection molding
PP	Polypropylene
PSD	Particle size distribution
pvT	Pressure-Volume-Temperature
PW	Paraffin Wax
SW	Synthetic Wax
SA	Stearic acid
TGA	Thermo gravimetric analysis
φ	Powder loading
φ_max_	Maximum powder loading
η	Shear Viscosity
φ_0_	Zero Shear Viscosity
α_STV_	Moldability
$\dot{\gamma }$	Shear rate
E	Activation energy for flow
R	Universal gas constant
T	Temperature
K	Constant of H-B model
n	Shear sensitivity
τ_y_	Yield Stress
S_W_	Width of the distribution
